# Evaluation of 41 Candidate Gene Variants for Obesity in the EPIC-Potsdam Cohort by Multi-Locus Stepwise Regression

**DOI:** 10.1371/journal.pone.0068941

**Published:** 2013-07-12

**Authors:** Sven Knüppel, Klaus Rohde, Karina Meidtner, Dagmar Drogan, Hermann-Georg Holzhütter, Heiner Boeing, Eva Fisher

**Affiliations:** 1 Department of Epidemiology, German Institute of Human Nutrition Potsdam-Rehbruecke, Nuthetal, Germany; 2 Exp. Genetics of Cardiovascular Diseases, Max Delbrück Center for Molecular Medicine Berlin-Buch, Berlin, Germany; 3 Department of Molecular Epidemiology, German Institute of Human Nutrition Potsdam-Rehbrücke, Nuthetal, Germany; 4 Institute of Biochemistry, Charité-Universitätsmedizin Berlin, Berlin, Germany; 5 Administrative Office of the Commission on Genetic Testing, Robert Koch-Institute, Berlin, Germany; Sanjay Gandhi Medical Institute, India

## Abstract

**Objective:**

Obesity has become a leading preventable cause of morbidity and mortality in many parts of the world. It is thought to originate from multiple genetic and environmental determinants. The aim of the current study was to introduce haplotype-based multi-locus stepwise regression (MSR) as a method to investigate combinations of unlinked single nucleotide polymorphisms (SNPs) for obesity phenotypes.

**Methods:**

In 2,122 healthy randomly selected men and women of the EPIC-Potsdam cohort, the association between 41 SNPs from 18 obesity-candidate genes and either body mass index (BMI, mean = 25.9 kg/m^2^, SD = 4.1) or waist circumference (WC, mean = 85.2 cm, SD = 12.6) was assessed. Single SNP analyses were done by using linear regression adjusted for age, sex, and other covariates. Subsequently, MSR was applied to search for the ‘best’ SNP combinations. Combinations were selected according to specific AIC_c_ and p-value criteria. Model uncertainty was accounted for by a permutation test.

**Results:**

The strongest single SNP effects on BMI were found for TBC1D1 rs637797 (β = −0.33, SE = 0.13), FTO rs9939609 (β = 0.28, SE = 0.13), MC4R rs17700144 (β = 0.41, SE = 0.15), and MC4R rs10871777 (β = 0.34, SE = 0.14). All these SNPs showed similar effects on waist circumference. The two ‘best’ six-SNP combinations for BMI (global p-value = 3.45⋅10^–6^ and 6.82⋅10^–6^) showed effects ranging from −1.70 (SE = 0.34) to 0.74 kg/m^2^ (SE = 0.21) per allele combination. We selected two six-SNP combinations on waist circumference (global p-value = 7.80⋅10^–6^ and 9.76⋅10^–6^) with an allele combination effect of −2.96 cm (SE = 0.76) at maximum. Additional adjustment for BMI revealed 15 three-SNP combinations (global p-values ranged from 3.09⋅10^–4^ to 1.02⋅10^–2^). However, after carrying out the permutation test all SNP combinations lost significance indicating that the statistical associations might have occurred by chance.

**Conclusion:**

MSR provides a tool to search for risk-related SNP combinations of common traits or diseases. However, the search process does not always find meaningful SNP combinations in a dataset.

## Introduction

Obesity is an increasing health problem worldwide that is associated with an increased risk of several common diseases including cardiovascular diseases, type 2 diabetes mellitus and certain cancers. The World Health Organization estimated that by 2008, 1.4 billion adults, 20 years and older, were overweight and from those more than 200 million men and nearly 300 million women were obese [1]. Although it is well known, that environmental and genetic factors contribute to the development of obesity, the genetic factors predisposing to obesity are still poorly understood [2]. Several studies identified a large number of single nucleotide polymorphisms (SNPs) as determinants of body mass index (BMI, kg/m^2^), waist circumference, and body fat mass as reviewed in Rankinen et al. [3]. More recently, large scale genome-wide association studies have led to additional discoveries of common obesity-related SNPs [4], [5]. However, one of the strongest common genetic predictor of body mass index, the genetic variants of the FTO gene (fat mass and obesity associated gene), explain only 1% of the total heritability of obesity [6].

So far, there is limited data about the extent to which non-additive effects of genes, mostly described in terms of gene–gene interaction, will add to the inherited risk for obesity development. It is generally assumed that several loci could interactively contribute to common diseases or traits with higher magnitude of effects than the single variants. Resolving such combined effects is imperative to enable the identification of persons at high risk based on their genetic profile.

In order to design a multi-locus based statistical tool to identify SNP combinations we extended the classical haplotype-based approach [7], [8] by combining it with stepwise regression [9] and applied this approach before to SNPs related to atopic dermatitis in a chromosomal region [10].

The aim of this study was to introduce an adapted version of the multi-locus stepwise regression (MSR) to combine SNP alleles from various chromosomes, i.e. unphased genotypes, in the way haplotypes are constructed [10], [11] and use those allele combinations as units for association analysis with a continuous outcome to identify particular allele combinations related to quantitative disease phenotypes. As an empirical example, we assessed the impact of allelic combinations derived from 41 candidate gene SNPs for obesity-related phenotypes (BMI and waist circumference) in a German population-based sample of healthy middle-aged men and women [12].

## Materials and Methods

### Ethics Statement

Written informed consent was obtained from all study participants, and approval was given by the Ethical Committee of the Medical Association of the State of Brandenburg, Germany.

### Subjects and Study Design

The European Prospective Investigation into Cancer and Nutrition (EPIC)-Potsdam cohort study is part of a large multi-center European-wide cohort study [13]. Recruitment of 27,548 participants, aged mainly 35 to 65 years, from the general population living in the area of Potsdam in northern Germany was conducted between 1994 and 1998. The baseline examination included anthropometric and blood pressure measurements, blood sampling, a self-administered validated food-frequency questionnaire, and a personal interview on lifestyle habits and medical history [12]. Baseline mean BMI was >25 kg/m^2^ (overall: 26.3±4.4 (mean±SD); women (n = 16,644): 25.8±4.7, and men (n = 10,904): 27.0±3.7). A random sample of 2,500 participants (62% women, 38% men) was selected from those participants who had provided blood samples at baseline (n = 26,664). From this representative ‘sub-cohort’ sample, participants with a history of diabetes, cancer, myocardial infarction, or stroke and those with missing relevant baseline information were excluded from the analysis. Hence, for the final analysis on anthropometric parameters data from 2,122 participants were used.

### Anthropometry Measurements

Weight, height, and waist-circumference were measured by trained interviewers with standardized methods described elsewhere [14]. Waist circumference was assessed by midway measurements between the lower rib margin and the superior anterior iliac spine to the nearest 0.5 cm. Body mass index was calculated by weight (kg) divided by squared height (m).

### Candidate Genes and Single Nucleotide Polymorphism (SNP) Selection

SNPs identified in genome wide association studies of body mass index or obesity (INSIG2 rs7566605 [15], FTO rs9939609 [16], MC4R rs17700144 [17], TMEM18 rs11127485 [17], NPC1 rs1805081 [18]), reported to be associated with body weight, body mass index, body composition indices (fat mass, fat-free mass, percentage body fat or sum of skinfolds), body fat distribution indices (waist circumference, waist-to-hip ratio, abdominal visceral and subcutaneous fat), leanness or obesity in human populations (MTTP rs3816873 [3], SREBF1 rs2297508 [19], LEPR (rs1137100 [3], rs1137101 [3], rs8179183 [3]), PPARG rs1801282 [3], FABP2 (rs6857641 [3], rs1799883 [3]), FABP1 rs2241883 [20], TCF7L2 rs7903146 [3], PTGES2 rs132823456 [21]) or located within quantitative trait loci (QTL) which constitute potential candidates influencing obesity-related phenotypes (ABCC8 [22], HSD11B1 [23], ALPI [24], IGF1 [25], TBC1D1 [26], M4CR [27], [28]) were included in the statistical modeling.

The majority of the selected SNPs were unlinked - each SNP represented one gene locus. Exceptions were made for FABP2 (two known functional SNPs), HSD11B1 (three SNPs), LEPR (three SNPs), MC4R (two SNPs), ABCC8 (six SNPs) and TBC1D1 (thirteen SNPs). The ATP-binding cassette transporter sub-family C member 8 gene (ABCC8), formerly known as pancreatic sulfonylurea receptor gene (SUR1), consists of ∼84 kilobases encompassing 39 exons that encode a protein of 1581 amino acids length [29]. The TBC1 domain family, member 1 (TBC1D1) gene (∼248 kilobases) encodes a protein consisting of 763 amino acid residues [29]. Both genes harbour a large number of SNPs (ABCC8: ∼1800 NCBI SNPs, TBC1D1: ∼4400 NCBI SNPs) [29]. Six SNPs from ABCC8 and thirteen SNPs from TBC1D1, tagging some of the genetic variation in both genes, were selected. LD maps of the respective SNPs of the ABCC8 and TBC1D1 genes are shown in Figures S1 and S2.

### Genotyping and Quality Control of the Data

Genotyping was performed before 2009 using TaqMan primers and technology (ABI, Forster City, CA, USA) as described previously [30]. The overall concordance rate was 98.8% based on 144 duplicates.

### Statistical Analysis

Genotype data of 41 SNPs in 18 candidate genes (ABCC8, ALPI, FABP1, FABP2, FTO, HSD11B1, IGF1, INSIG2, LEPR, MC4R, MTTP, NPC1, PPARG, PTGES2, SREBF1, TBC1D1, TCF7L2, TMEM18) distributed among 11 chromosomes were categorized into three groups (homozygote major allele, heterozygote, and homozygote minor allele). Single SNP effects on BMI and waist circumference were tested using a multiple linear regression model that assumed an additive effect by modeling the number of minor alleles (0 = homozygotes major allele, 1 = heterozygous, and 2 = homozygous minor allele).

For estimating the effect of single SNPs and of the combined multiple SNPs (SNP combinations), the statistical model was adjusted for commonly known obesity-related factors such as sex, age at baseline, educational attainment (no degree/primary school, Technical/professional school, Secondary school, and University degree), occupational activity (light, moderate, heavy), sports activity (0, <4, >4 h/week), smoking habits (never, past, current), alcohol intake (0, <5, 5–10, 10–20, 20–40, >40 g/day), total daily energy (kJ/day), fat intake (g/day), and fruit and vegetable intake (g/day). Furthermore, the genetic effect on abdominal obesity was assessed by adjusting the models on waist circumference for BMI.

Haplotype analysis was done for the six genes which comprised two or more SNPs (LEPR, HSD11B1, TBC1D1, FABP2, ABCC8, and MC4R). Haplotypes were estimated by the expectation-maximization (EM) algorithm [31] and evaluated separately using F test. Single haplotypes with frequency >0.05 were tested by comparing the model including the estimated individual probabilities for one haplotype and the covariates against the model excluding the single haplotype estimates.

SNP-sex interaction was tested by including a SNPxSEX interaction term in the adjusted models. The interaction p-values were determined by F test comparing the model including all covariates, SNP, sex, and the interaction term against the model excluding the interaction term.

Statistical evaluation of SNP combinations was conducted by multi-locus stepwise regression (MSR) [10], [11]. MSR combines the concept of haplotype association testing [7], [8] and the classical stepwise regression approach [9] to select subsets of SNPs with a high impact on the phenotype.

The principle of MSR can be summarized as follows. The methods developed for haplotypes were used in the same way for unlinked genetic markers. SNP allele combinations (multi-locus marker) instead of haplotypes were tested. Allele combinations with a frequency less than 5% were rated as “rare” and subsequently pooled to avoid spurious associations due to low frequencies. Starting point was a pair-wise SNP association-test where all possible two-SNP allele combinations were tested for their association with the quantitative traits using multiple linear regression models. The individual probabilities of the SNP allele combinations consistent with the observed genotypes were estimated using the expectation-maximization (EM) algorithm [31]. To evaluate the overall fit, we used the global F-test comparing the model including covariates and all allele combinations against the model including only covariates. The results of all pair-wise SNP global F-tests were then sorted in ascending order by p-value. All SNP-pairs with a p-value lower than 0.05 were selected. These SNP combinations were labeled as ‘best’ two-SNP combinations. Next, the impact of all three-SNP combinations was modeled from the best SNP-pairs by adding one SNP out of the remaining SNPs and using again the EM algorithm to estimate individual probabilities for their allele combinations [31]. Those three-SNP combinations which showed an improvement of the corrected Akaike’s information criterion AIC_c_ 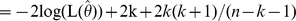, where 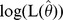 is the maximum likelihood estimate of the model, k denotes the number of model parameters, and n denotes the sample size [32], were further processed and tested for association by the global F-test as described before. Next, the impact of all four-SNP combinations was assessed in the same way by extension of the best three-SNP combinations (p<0.01) with one of the remaining SNPs. As before, the new SNP combinations were only considered for further processing if the AIC_c_ values showed an improvement and the p-value was below a certain threshold. The thresholds used for every step were 0.05, 0.01, 0.001, and 0.0001 for SNP combinations of two to five SNPs, respectively. The procedure can be repeated until a pre-defined number of maximum SNPs per combination is reached or no more SNP combinations fulfill the AIC_c_ and p-value criteria.

The effect of each single allele combination included in the best selected SNP combinations were assessed by comparing the results from the multiple linear regression models including each allele combination and all covariates against the model including only covariates. We used the F test to determine the statistical significance for each single allele combination, separately.

Each allele combination is characterized by the combination of major (coded as 1) or minor alleles (coded as 2) of the included SNPs (loci). For instance, the allele combination 112111 (the first six-SNP combination derived from MSR for BMI) consists at the first given two loci of the major alleles, followed by one minor allele and at the last given three loci of the major alleles. All individual genotypes could be assigned with pairs of such allele combinations weighted by their estimated frequencies. Each individual genotype is compatible to none, one (heterozygous), or pairs (homozygous) of such allele combinations. The beta-coefficient for that allele combination of −1.70 kg/m^2^ means a prediction of a 1.70 kg/m^2^ lower average of BMI for individuals which were heterozygous for that allele combination compared to individuals which had a genotype which was not comparable to that allele combination. If an individual is homozygous for that allele combination, then the expected mean of BMI would be 2⋅1.70 = 3.40 kg/m^2^ lower than individuals carrying none of this allele combination.

The effect of model uncertainty was assessed by using a resampling method [33]. We used a permutation approach in order to relax the assumption of independency of the test statistics and to derive the distribution under the null hypothesis. The permutation approach was used to evaluate the results of the best SNP combinations.

Using the permutation approach we derived the distribution of the test statistic under the null hypothesis by random permutations of the trait values and holding the non-genetic covariates constant relative to the trait values. In each permutation the SNP combinations remained unchanged. In this permutation approach the phenotype-covariate-association was held constant by keeping the covariates for each trait value. We focused on the relationship between the phenotype and the SNP combinations but maintained the relationship within the SNP combinations. These permutation samples are assumed to represent the distribution under the null hypothesis. The permutation-based p-value was calculated as proportion of the lowest p-value of each permutation sample less than the observed p-value.

The effect of random selection of the best SNP combinations was assessed by using 200 permutations and running the MSR for each permutation. The permutations could result in SNP combinations with different lengths, i.e. number of combined SNP alleles, and different SNPs. The lowest p-value of each permutation was stored, and results were compared based on the original sample with the distribution of the lowest p-values derived by the permutation approach.

### Simulation Study

We evaluated the chance of “random selection” by a small simulation study (type I error). Assuming no genetic effect we simulated 41 unlinked SNPs with equal minor allele frequencies of 0.2 for 2,156 individuals. Each individual was assigned to a random phenotype value taken from a normal distribution with mean = 25.9 and standard deviation = 4.1, which equaled the average BMI value in our study. This procedure was repeated 1,000 times and within each replication we applied MSR. For simplicity and since the simulation study was small, we only used the p-value for interpretation.

The Statistical Software R (version 2.14.0) [34] was Applied for the Analyses.

## Results

A description of baseline characteristics of the study sample for this analysis is given in Table 1. 1,320 of 2,122 study participants (62%) were women with mean age of 48.0 (SD = 9.1). Men (38%) were 51.1 years (SD = 8.0) on average. The average BMI of both, men (26.6 kg/m^2^, SD = 3.4) and women (25.5 kg/m^2^, SD = 4.5), were slightly above the threshold for “normal” weight. The mean waist circumference was higher for men (93.6 cm, SD = 9.7) than for women (80.1 cm, SD = 11.3). Further, the participants were predominantly highly educated and less active. Most of the participants were nonsmokers (48% never and 32% former smoker) and drank less than 40 g/day alcohol (81%). No alcohol was consumed by 3% of the participants. Mean energy (men: 10,748 kJ/day, women: 7,409 kJ/day), fat, (men: 117.8 g/day, women: 75.2 g/day) and fruit and vegetable intake (268.7 g/day, women: 306.6 g/day) differed between men and women.

**Table 1:** Characteristics of study participants at baseline in the EPIC-Potsdam subsample.

	Total	Men	Women
**Sex, N (%)**	2,122 (100)	802 (37.8)	1,320 (62.2)
**Age, years, mean (sd)**	49.2 (8.8)	51.1 (8.0)	48.0 (9.1)
**BMI, kg/m^2^, mean (sd)**	25.9 (4.1)	26.6 (3.4)	25.5 (4.5)
**Height, cm, mean (sd)**	167.7 (8.5)	175.2 (6.5)	163.1 (6.0)
**Waist circumference, cm, mean (sd)**	85.2 (12.6)	93.5 (9.7)	80.1 (11.3)
**Waist-Hip-Ratio, mean (sd)**	0.85 (0.1)	0.94 (0.1)	0.79 (0.1)
**Education, N (%)**			
No degree/primary school	67 (3.2)	14 (1.7)	53 (4.0)
Technical/professional school	708 (33.4)	238 (29.7)	470 (35.6)
Secondary school	519 (24.5)	122 (15.2)	397 (30.1)
University degree	828 (39.0)	428 (53.4)	400 (30.3)
**Occupational activity, N (%)**			
Light	1,272 (59.9)	456 (56.9)	816 (61.8)
Moderate	722 (34.0)	260 (32.4)	462 (35.0)
Heavy	128 (6.0)	86 (10.7)	42 (3.2)
**Sport activity, N (%)**			
No physical activity	1,244 (58.6)	504 (62.8)	740 (56.1)
<4 h/week	767 (36.1)	247 (30.8)	520 (39.4)
4+ h/week	111 (5.2)	51 (6.4)	60 (4.5)
**Smoking status, N (%)**			
Never	1,011 (47.6)	242 (30.2)	769 (58.3)
Past	675 (31.8)	351 (43.8)	324 (24.5)
Current	436 (20.5)	209 (26.1)	227 (17.2)
**Alcohol intake, N (%)**			
No ethanol intake	62 (2.9)	26 (3.2)	36 (2.7)
<5 g/day	731 (34.4)	116 (14.5)	615 (46.6)
5–10 g/day	411 (19.4)	105 (13.1)	306 (23.2)
10–20 g/day	399 (18.8)	169 (21.1)	230 (17.4)
20–40 g/day	328 (15.5)	223 (27.8)	105 (8.0)
40+ g/day	191 (9.0)	163 (20.3)	28 (2.1)
**Energy intake, kJ/day, mean (sd)**	8,671 (2738)	10,748 (2827)	7,409 (1728)
**Fat intake, g/day, mean (sd)**	91.3 (37.6)	117.8 (41.5)	75.2 (23.1)
**Fruit and vegetable intake, g/day, mean (sd)**	292.3 (137.2)	268.7 (131.9)	306.6 (138.4)

SD = Standard deviation.

### Single Marker Analyses (SNP Effects)

All SNPs had a minor allele frequency (MAF) of at least 5% and passed the Hardy-Weinberg equilibrium (HWE) test (exact HWE p-value cut off ≥0.001). The average missing genotype proportion was 2.1%. The obtained MAFs were consistent with those reported in the HapMap CEU data of European descent.

Nominal significant effects on BMI were found for TBC1D1 rs637797 (ß = −0.33, SE = 0.13, p = 0.013), FTO rs9939609 (ß = 0.28, SE = 0.13, p = 0.026), MC4R rs17700144 (ß = 0.41, SE = 0.15, p = 0.006), and MC4R rs10871777 (ß = 0.34, SE = 0.14, p = 0.017). TBC1D1 rs637797 (ß = −0.85, SE = 0.34, p = 0.0012) and MC4R SNPs (ß_rs17700144_ = 1.11, SE = 0.38, p = 0.003 and ß_rs10871777_ = 0.91, SE = 0.36, p = 0.012) showed also an association with waist circumference. Nominal significant effect on waist circumference was observed for IGF1 rs1520220 (ß = 0.81, SE = 0.40, p = 0.042) and a similar effect for PPARG rs1801282 (ß = 0.75, SE = 0.43, p = 0.082). Nominal significant effect on waist circumference adjusted for BMI was found for ABCC8 rs10832786 (ß = −0.47, SE = 0.20, p = 0.022). All single SNP associations did not withstand corrections for multiple testing (Table 2).

**Table 2:** SNP characteristics and associations of SNP alleles with obesity-related traits in 2,122 European middle-aged men and women (random population sample).

ID	SNP	Gene	Chr	Position	Minor/major	Missing	MAF	HWE p^a^	BMI(kg/m^2^)^b^		WC (cm)^b^		WC (cm)adj. BMI b	
					allele				Beta (SE)	p	Beta (SE)	p	Beta (SE)	p
1	rs1137100	LEPR	1	65809029	A/G	2%	0.26	0.367	−0.17 (0.14)	0.214	−0.64 (0.35)	0.071	−0.25 (0.17)	0.133
2	rs1137101	LEPR	1	65831101	G/A	2%	0.46	0.402	0.01 (0.12)	0.946	−0.26 (0.31)	0.401	−0.28 (0.14)	0.051
3	rs8179183	LEPR	1	65848540	G/C	1%	0.16	0.339	−0.11 (0.16)	0.492	−0.13 (0.42)	0.756	0.13 (0.19)	0.517
4	rs4844880	HSD11B1	1	207937539	T/A	0%	0.17	0.074	0.20 (0.17)	0.222	0.64 (0.43)	0.134	0.18 (0.20)	0.367
5	rs846910	HSD11B1	1	207941877	G/A	1%	0.05	0.827	−0.09 (0.27)	0.738	−0.25 (0.70)	0.726	−0.04 (0.32)	0.909
6	rs3753519	HSD11B1	1	207942138	G/A	1%	0.11	0.830	0.10 (0.19)	0.606	0.33 (0.50)	0.509	0.10 (0.23)	0.661
7	rs11127485	TMEM18	2	622028	T/C	3%	0.17	0.538	−0.04 (0.17)	0.793	−0.26 (0.42)	0.544	−0.16 (0.20)	0.419
8	rs2241883	FABP1	2	88205181	T/C	1%	0.37	0.063	−0.11 (0.12)	0.370	−0.36 (0.32)	0.257	−0.11 (0.15)	0.464
9	rs7566605	INSIG2	2	118552495	G/C	1%	0.31	0.686	−0.05 (0.13)	0.675	−0.01 (0.34)	0.972	0.11 (0.16)	0.463
10	rs3762521	ALPI	2	233026468	A/G	1%	0.28	0.030	0.00 (0.13)	0.977	−0.04 (0.34)	0.913	−0.03 (0.16)	0.857
11	rs1801282	PPARG	3	12368125	C/G	1%	0.16	0.805	0.26 (0.17)	0.121	0.75 (0.43)	0.082	0.16 (0.20)	0.430
12	rs2279027	TBC1D1	4	37580151	G/A	1%	0.41	0.115	0.07 (0.12)	0.578	0.20 (0.31)	0.517	0.05 (0.15)	0.738
13	rs35859249	TBC1D1	4	37580484	C/T	0%	0.08	0.544	−0.19 (0.23)	0.400	−0.40 (0.58)	0.494	0.04 (0.27)	0.891
14	rs4832743	TBC1D1	4	37601611	T/C	1%	0.36	0.322	−0.03 (0.13)	0.785	−0.27 (0.32)	0.408	−0.19 (0.15)	0.204
15	rs10517456	TBC1D1	4	37631127	A/G	1%	0.45	0.509	−0.03 (0.12)	0.839	0.20 (0.32)	0.530	0.26 (0.15)	0.079
16	rs9999507	TBC1D1	4	37677567	A/G	1%	0.50	0.116	−0.03 (0.12)	0.821	−0.26 (0.31)	0.401	−0.20 (0.14)	0.168
17	rs6845120	TBC1D1	4	37723940	A/G	1%	0.29	0.958	0.04 (0.13)	0.741	−0.13 (0.34)	0.700	−0.23 (0.16)	0.142
18	rs6823014	TBC1D1	4	37730977	A/G	2%	0.50	1.000	−0.17 (0.12)	0.168	−0.19 (0.32)	0.554	0.20 (0.15)	0.171
19	rs10009706	TBC1D1	4	37755870	A/G	2%	0.26	0.910	0.03 (0.14)	0.804	−0.06 (0.36)	0.863	−0.14 (0.17)	0.394
20	rs2303422	TBC1D1	4	37768195	T/C	2%	0.16	0.739	0.09 (0.17)	0.610	−0.06 (0.43)	0.895	−0.25 (0.20)	0.208
21	rs1344603	TBC1D1	4	37786242	C/T	1%	0.32	0.724	0.02 (0.13)	0.883	0.20 (0.34)	0.552	0.16 (0.16)	0.316
22	rs637797	TBC1D1	4	37802493	T/A	1%	0.30	0.571	−0.33 (0.13)	0.013	−0.85 (0.34)	0.012	−0.11 (0.16)	0.493
23	rs6837834	TBC1D1	4	37810380	T/C	1%	0.12	0.015	0.18 (0.18)	0.341	0.34 (0.48)	0.479	−0.06 (0.22)	0.770
24	rs13110318	TBC1D1	4	37815251	G/A	1%	0.08	0.663	−0.24 (0.22)	0.279	−0.66 (0.58)	0.248	−0.11 (0.27)	0.673
25	rs3816873	MTTP	4	100723687	T/C	1%	0.25	0.357	−0.05 (0.14)	0.695	−0.09 (0.35)	0.810	0.04 (0.16)	0.818
26	rs1799883	FABP2	4	120461350	G/A	1%	0.26	0.776	−0.23 (0.14)	0.096	−0.35 (0.36)	0.332	0.18 (0.17)	0.275
27	rs6857641	FABP2	4	120462959	C/T	2%	0.43	0.212	−0.19 (0.12)	0.118	−0.45 (0.31)	0.151	−0.02 (0.14)	0.917
28	rs13283456	PTGES2	9	129924574	C/T	2%	0.18	0.496	0.10 (0.16)	0.544	0.26 (0.41)	0.525	0.04 (0.19)	0.833
29	rs7903146	TCF7L2	10	114748339	C/T	0%	0.26	0.279	−0.15 (0.14)	0.273	−0.41 (0.36)	0.244	−0.07 (0.16)	0.678
30	rs916829	ABCC8	11	17397049	C/T	0%	0.15	0.671	−0.24 (0.17)	0.153	−0.36 (0.43)	0.409	0.19 (0.20)	0.338
31	rs916828	ABCC8	11	17397356	C/G	1%	0.27	0.781	−0.09 (0.14)	0.522	0.00 (0.36)	0.994	0.20 (0.16)	0.224
32	rs2237984	ABCC8	11	17402597	G/A	1%	0.39	0.110	−0.07 (0.12)	0.577	−0.35 (0.32)	0.270	−0.19 (0.15)	0.188
33	rs10832786	ABCC8	11	17403538	T/A	1%	0.14	0.128	0.24 (0.17)	0.162	0.08 (0.44)	0.849	−0.47 (0.20)	0.022
34	rs7106053	ABCC8	11	17415306	G/A	1%	0.34	0.698	0.03 (0.13)	0.792	−0.07 (0.33)	0.843	−0.14 (0.15)	0.352
35	rs11024286	ABCC8	11	17415683	G/A	1%	0.31	0.919	−0.01 (0.13)	0.968	−0.12 (0.34)	0.722	−0.11 (0.16)	0.489
36	rs1520220	IGF1	12	101320652	C/G	2%	0.18	0.560	0.29 (0.16)	0.060	0.81 (0.40)	0.042	0.15 (0.19)	0.436
37	rs9939609	FTO	16	52378028	T/A	1%	0.42	0.165	0.28 (0.13)	0.026	0.59 (0.32)	0.068	−0.05 (0.15)	0.740
38	rs2297508	SREBF1	17	17656042	G/C	4%	0.37	0.004	0.16 (0.13)	0.193	0.38 (0.32)	0.237	0.01 (0.15)	0.955
39	rs1805081	NPC1	18	19394430	A/G	3%	0.42	0.391	−−0.02 (0.12)	0.888	−0.14 (0.32)	0.669	−0.10 (0.15)	0.512
40	rs17700144	MC4R	18	55962962	G/A	2%	0.21	0.017	0.41 (0.15)	0.006	1.11 (0.38)	0.003	0.18 (0.18)	0.312
41	rs10871777	MC4R	18	56002743	A/G	2%	0.23	0.027	0.34 (0.14)	0.017	0.91 (0.36)	0.012	0.14 (0.17)	0.396

ID = identification number, WC = waist circumference, MAF = minor allele frequency, HWE = Hardy Weinberg equilibrium, SE = Standard error, N = Number of individuals.

a p-value of exact HWE test.

b Results of multiple linear regression analyses adjusted for sex, age, education, occupational activity, smoking, and alcohol, energy, fat, fruit and vegetable intake. SNPs were included in the models coded as 0, 1, 2 for minor allele count.

### Haplotypes

We applied haplotype analysis on SNPs in LEPR, HSD11B1, TBC1D1, FABP2, ABCC8, and MC4R. None of the tested haplotypes showed robust effects with exception of MC4R on BMI and waist circumference which showed the same effect as the single-loci analysis (Tables S1–3). For example, the haplotype 11 consisting of the major allele on both MC4R-SNPs showed an effect of ß = −0.34 kg/m^2^ (SE = 0.14, p = 0.015, frequency = 0.77) on BMI.

### SNP-sex Interaction

Five SNP-sex interaction terms resulted in p-values lower than 0.05, but would not survive a correction for multiple testing (Table S4): PPARG rs1801282 (p = 0.03 for BMI and p = 0.03 for waist circumference), TBC1D1 rs637797 (p = 0.04, BMI), and FTO rs9939609 (p = 0.03 for waist circumference and p = 0.03 for waist circumference adjusted for BMI).

### Multi-Locus Stepwise Regression (MSR) Analyses

Associations of SNP combinations with BMI, waist circumference, and waist circumference adjusted for BMI were analyzed using MSR. Starting with two-SNP combinations we progressively increased the number of SNPs. In each step, statistical significance of the global F-test increased approximately by a factor of 10 (Table S5), e.g. for BMI the mean of p-values decreased from 2.62⋅10^–2^ (two-SNP combinations) to 5.43⋅10^–4^ (four-SNP combinations) and 7.71⋅10^–6^ (six-SNP combinations). Decreased p-values were also observed for the two search processes on waist circumference (Table S5). MSR stopped at three-SNP combinations for waist circumference adjusted for BMI. By adding one SNP at a time in each step, heterogeneity of the derived SNP combinations between individuals increased and their frequencies decreased, e.g. the mean of allele combination frequencies were 28% (two-SNP combinations), 13% (four-SNP combinations), and 10% (six-SNP combinations) for BMI.

Table 3 shows the first two results for the SNP combinations having met the AIC_c_ and the p-value threshold criteria for each phenotype. Nine combinations of six SNPs were identified as best SNP combinations associated with BMI. Two six-SNP combinations were identified for waist circumference and fifteen three-SNP combinations for waist circumference adjusted for BMI. The construction process of MSR for each phenotype is shown in Tables S6–8.

**Table 3:** Associations of the MSR-selected SNP combinations with obesity-related traits in 2,122 European middle-aged men and women (random population sample).

Phenotype global p-value (F test) (permutation p-value)^a^	SNPs^b^	allele	Frequency	Beta (SE)^b^	p-value
		combination			
**BMI (kg/m^2^)**	4-19-26-36-37-40	112111	0.062	−1.70 (0.34)	9.56E-07
p = 3.45E-06 (0.38)		121111	0.064	−0.53 (0.33)	1.09E-01
		111111	0.168	−0.21 (0.20)	2.91E-01
		111121	0.131	0.10 (0.22)	6.63E-01
**BMI (kg/m^2^)**	7-13-15-18-29-30	111211	0.116	−0.28 (0.24)	2.36E-01
p = 6.82E-06 (0.49)		112111	0.099	−0.17 (0.26)	5.23E-01
		112211	0.116	0.58 (0.25)	1.84E-02
		111111	0.143	0.74 (0.21)	3.91E-04
**WC (cm)**	23-27-32-36-37-40	121111	0.094	−2.54 (0.69)	2.35E-04
p = 7.80E-06 (0.52)		122111	0.057	−2.40 (0.92)	9.22E-03
		112121	0.057	−2.22 (0.91)	1.53E-02
		121121	0.064	−0.83 (0.86)	3.36E-01
		112111	0.077	−0.73 (0.78)	3.52E-01
		111111	0.105	0.14 (0.66)	8.27E-01
		111121	0.084	1.50 (0.75)	4.53E-02
**WC (cm)**	4-27-32-36-37-40	121111	0.079	−2.96 (0.76)	1.08E-04
p = 9.76E-06 (0.54)		122111	0.057	−2.46 (0.90)	6.33E-03
		112121	0.053	−2.17 (0.95)	2.24E-02
		121121	0.066	−1.07 (0.84)	2.05E-01
		112111	0.073	−0.92 (0.81)	2.58E-01
		111111	0.103	0.21 (0.66)	7.50E-01
		111121	0.087	1.02 (0.73)	1.61E-01
**WC adj. BMI (cm)**	3-26-33	112	0.086	−1.11 (0.28)	9.51E-05
p = 3.09E-04 (0.83)		211	0.108	−0.01 (0.26)	9.79E-01
		121	0.184	0.06 (0.20)	7.65E-01
		111	0.531	0.16 (0.15)	2.84E-01
**WC adj. BMI (cm)**	5-26-33	112	0.098	−0.96 (0.26)	2.22E-04
p = 2.78E-03 (0.96)		121	0.205	0.15 (0.19)	4.20E-01
		111	0.607	0.18 (0.15)	2.28E-01

Association results are based on models adjusted for sex, age, education, occupational activity, sports activity, smoking, and alcohol, energy, fat, fruit and vegetable intake. SE = standard error.

a Permutation test based on 200 permutations.

b SNP ID numbers according to Table 2.

c The major allele was coded as 1 and the minor one as 2.

The best SNP combinations for BMI showed two different patterns. The first pattern included the SNPs FABP2 rs1799883, FTO rs9939609, and one of the two MC4R-SNPs. Further SNPs were mutually interchanged: HSD11B1 rs4844880, PPARG rs1801282, TBC1D1 rs6845120, TBC1D1 rs10009706, ABCC8 rs10832786, and IGF1 rs1520220. The second pattern contained TBC1D1 rs35859249, TBC1D1 rs10517456, TBC1D1 rs6823014, and TCF7L2 rs7903146. Further SNPs were mutually interchanged: LEPR rs11371001, TMEM18 rs11127485, ABCC8 rs916829, ABCC8 rs916828, and SREBF1 rs2297508.

The two SNP combinations for waist circumference were very similar. They differed only in the first position where HSD11B1 rs4844880 and TBC1D1 rs6837834 were interchanged. Both SNP combinations contained FABP2 rs6857641, ABCC8 rs2237984, IGF1 rs1520220, FTO rs9939609, and MC4R rs17700144.

All but one of the best SNP combinations for waist circumference adjusted for BMI did not contain SNP ABCC8 rs10832786 which had shown a nominal significant single SNP effect on waist circumference adjusted for BMI. The first four SNP combinations also contained FABP2 rs1799883 and additionally LEPR rs8179183 or HSD11B1 rs846910.

Among all identified SNP combinations at least one allele combination had a higher effect size than those found in single SNP associations. For BMI and waist circumference the identified SNP combinations contained SNPs which were also nominally significant associated in the single SNP analyses (for BMI: FTO rs9939609 and both MC4R-SNPs). FTO rs9939609 was also present in the two selected SNP combinations related to waist circumference but not in the best SNP combinations for waist circumference adjusted for BMI.

For the best six-SNP combination identified for BMI, the four most common allele combinations showed frequencies of 0.168, 0.131, 0.064, and 0.062, respectively (Table 3). The second best six-SNP combination showed four allele combinations with frequencies of 0.143, 0.116, 0.116, and 0.099, respectively. The SNP combinations related to waist circumference showed more heterogeneity. Both best six-SNP combinations were represented by six allele combinations. The frequencies ranged from 0.05 to 0.10 among those variants. The maximum sum of allele combination frequencies (54%) for the selected SNP-combinations on BMI and waist circumference was found for the best six-SNP combination on waist circumference indicating that the aggregated ‘rare’ allele combinations (<5%) together summed to 46%. As expected, we found several low frequency allele combinations.

The effect sizes for BMI ranged from −1.70 kg/m^2^ (SE = 0.34) per allele combination to 0.74 kg/m^2^ (SE = 0.21) (Table 3). Compared to that, the strongest effect size of the single SNPs was observed for MC4R rs17700144 with 0.41 kg/m^2^ (SE = 0.15). The effects on waist circumference not adjusted for BMI were stronger compared to the effects on waist circumference adjusted for BMI. The strongest effect of allele combinations on waist circumference was −2.96 cm (SE = 0.76) and on waist circumference adjusted for BMI was −1.26 cm (SE = 0.36) (Table S9).

However, permutation testing to evaluate the effect of random selection of the MSR under the null hypothesis of no effect showed that all of the constructed permutation p-values for each phenotype were higher than the commonly used threshold of 5% (Table 3). All identified SNP combinations therefore would be classified as non-significant indicating a high likelihood of random selection.

### Simulation Study

On average 5% of the single SNP p-values from each MSR replication were lower than 0.05 indicating that the simulation process might have been successful because we modeled no genetic effects. The minimal p-value of each MSR run ranged from 1.5⋅10^–12^ to 3.5⋅10^–2^ with 5% percentile of 5.6⋅10^–9^ and median p-value of 4.9⋅10^–6^.

## Discussion

We applied a multi-locus stepwise regression approach on 41 SNPs from known candidate gene loci for obesity traits to find trait-related SNP combinations in a population-based study sample, consisting of >2000 middle-aged participants of the EPIC-Potsdam cohort. As starting point we found some candidate gene SNPs to be nominally associated with BMI, waist circumference, and waist circumference adjusted for BMI including for instance FTO rs9939609 (previously published in [35]) and two MC4R SNPs (previously published in [27]). The observed effect sizes were moderate, e.g. −0.33 kg/m^2^ for BMI and −0.85 cm for waist circumference per minor allele of TBC1D1 rs637797, respectively. Furthermore, we observed no haplotype effects in five genes (LEPR, HSD11B1, TBC1D1, FABP2, and ABCC8) and in MC4R two haplotypes showed similar effects as found in the single SNP tests.

Following our hypothesis that combinations of candidate gene SNPs might explain a larger proportion of the heritability of obesity-related anthropometric phenotypes we applied multi-locus stepwise regression (MSR) to identify trait-related SNP combinations. MSR revealed several low-frequency allele combinations (frequencies range: 5% to 23%) for BMI and waist circumference within those SNP combinations which showed a considerably higher impact on the obesity-related anthropometric phenotypes compared to single SNP associations. For example, the allele combination comprising the major alleles of HSD11B1 rs4844880, TBC1D1 rs10009706, IGF1 rs1520220, FTO rs9939609, MC4R rs17700144, and the minor allele of FABP2 rs1799883 was associated with a decrease of −1.70 kg/m^2^ BMI (nominal p-value = 9.56⋅10^–7^). Thus, this allele combination showed a remarkably large effect.

This study is hypothesis-driven since it utilizes prior information from genome-wide association, candidate gene and other studies. Given the high number of null hypotheses tested in the MSR, excessive adjusting of p-values for multiplicity could seriously hinder finding relevant genetic variants. However, too low restrictions within the stepwise search process would result in a magnitude of SNP combinations more likely to be false-positive. As Curtin et al. (2010) recommended for their algorithm implemented in hapConstructor we used stringent significance thresholds in each step of the MSR to focus on final SNP combinations which may not fail multiple testing corrections [11], [36].

Curtin et al. (2010) used the hapConstructor approach [11] to identify haplotypes within three genes (IKBKB, IL6, and NFKB1) associated with rectal and colorectal cancer. Furthermore, the authors searched for combined SNP effects across all three genes by building composite genotypes. They modeled also combinations of dominant and recessive SNP genotypes in a stepwise manner. They did not use allele dosis coding (0, 1, and 2) because they wanted to avoid sparse cells while modeling. However, to combine SNP genotypes is a general challenge because k SNP could be combined in 3^k^ possible genotypes. Depending on the minor allele frequencies many of the multi-SNP genotypes could be rare (frequencies <0.05). Therefore, the usage of genotypes is not as useful for combining many unlinked SNPs as the use of alleles.

In some respect, the MSR approach is similar to the hapConstructor approach [11]. Both approaches are based on stepwise selected haplotypes associated with diseases. Compared to hapConstructor, MSR uses the global test statistic for selection instead of single haplotypes. Selection strategy of the hapConstructor algorithm is based on p-values accounted for multiple testing in a Monte Carlo framework. In our study we used additionally to the p-value the AIC_c_ criterion as measurement of goodness–of-fit, thus taking account of the model complexity. We applied the MSR to find allele combinations associated with continuous traits instead using SNP genotypes associated with disease as done by Curtin et al. (2010).

Compared to the observed genotypes, allele combinations were inferred in each step of the MSR. Therefore, interpretations of such allele combinations should only be done on the background of all included SNPs and with care.

The correct number of independent test statistics for the application of Bonferroni- or other correction methods (e.g. FDR) while running MSR is unknown. The number of independent tests for correction lies somewhere between the number of actual conducted tests and the number of all possible tests up to n-SNP combinations n_combinations = 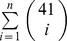
_, for instance if n = 6 we get 5,358,577 possible SNP combinations. However, these combinations are not independent because each SNP will be part of different SNP combinations.

Therefore, the model uncertainty of the selection strategy was evaluated by using a resampling method: The distribution under the null hypothesis was simulated by 200 permutations of the trait values holding the relationship between the phenotype and non-genetic covariates constant. It was shown that each of the permuted p-values of the best SNP combination were higher than the arbitrary significance level of 5% indicating that our result might have occurred by chance due to the high number of possible SNP combinations. Our finding points to a serious problem in a combination analysis. The selection strategy of unlinked marker combinations can yield significant false-positive effects due to the high number of possible combinations. The sole solution to overcome this problem is an independent replication in a second study sample, which was not available for our study.

The small simulation study showed that under the assumption of no genetic effect the simulated p-values are in the range of the p-values of the observed SNP combinations. This result also suggests that our selected SNP combinations are very likely to be a random finding. More intensive investigations on the feasibility and power of haplotype-based stepwise regression models for selecting SNP combinations of unlinked SNPs are needed.

Our study population consisted of a random sample representative of the general population of a distinct area. On average study participants were slightly overweight (BMI_men_ = 26.6, BMI_women_ = 25.5). The genetic effect was assessed on a continuous scale of obesity-related phenotypes and not restricted to obese individuals which may have attenuate our empirical results, i.e. in another study sample enriched with extreme phenotypes, associations might have been stronger.

We evaluated the presumable biological background of the found allele combinations by composing a simplified scheme (Text S1) of the physiological effects that the selected genes according to biochemical evidences may exert on the hypothalamic regulation of satiety (MC4R, LEPR), the transfer of lipids from the intestine to the blood plasma (MTP, FABP2) and the storage of lipids in the adipose tissue (NPC1, PPARG, ABCC8). Eventually, we noticed that the combinations of SNPs compiled by purely statistical criteria could not be directly brought in line with the developed scheme indicating the challenge to get deeper insights beyond single SNPs and their estimated combined effects as studied in this work.

It is a hypothetical assumption that in studies like this which aim to evaluate statistical epistasis, the identification of SNP combinations may lead to the recognition of unknown interactions as for example HSD11B1 rs4844880 showed no single marker effect on BMI, but was present in the best six-SNP combination for BMI in our study. The biological basis of statistical effect measurement modifications - if they were to be proved by replication - is, however, far more difficult to resolve. At this point, we wish to mention that a recent study by Zuk et al. [37] showed that ‘a substantial proportion’ of missing heritability - reasoned by the low impact of GWAS results - might well be attributed to underlying interactions between those variants identified so far. Thus, the proportion of missing heritability has been overestimated before. Especially, in obesity research the number and effect sizes of trait-related genetic variants have not yet reached the proportion of estimated inheritance by far. Therefore, efforts to elucidate unknown interactions between known candidate gene SNPs could lead to a better understanding in that field.

In our study, nominal significant associations of selected candidate-gene SNPs and multi-locus SNP combinations (derived from unphased genotype data) with obesity-related measurements (BMI and waist circumference) did not withstand multiple testing correction although some single variants and SNP combinations showed meaningful effects. We conclude that the use of systematic search procedures like MSR requires careful consideration of the search process in order to minimize the chance for false-positive findings. Resampling methods can be used to investigate such model uncertainties.

## Supporting Information

Figure S1 **LD plot for ABCC8 gene of the EPIC Potsdam subsample (2,122). Disequilibrium coefficient r^2^ values were generated using Haploview version 4.2 (Barrett JC et al. Bioinformatics 2005;21(2):263-5). Standard Color Scheme for the LD plots for r^2^ were used (for more information see Haploview documentation).**
(PDF)

Figure S2 **LD plot for TBC1D1 gene of the EPIC Potsdam subsample (N = 2,122). Disequilibrium coefficient r^2^ values were generated using Haploview version 4.2 (Barrett JC et al. Bioinformatics 2005;21(2):263-5). Standard Color Scheme for the LD plots for r^2^ were used (for more information see Haploview documentation).**
(PDF)

Table S1 **Single haplotype analysis on body-mass index (kg/m^2^) in the EPIC-Potsdam subsample (n = 2,122) with adjustment for sex, age at baseline, educational attainment, occupational activity, sports activity, smoking habits, alcohol intake, energy intake, fat intake, and fruit and vegetable intake.**
(PDF)

Table S2 **Single haplotype analysis on waist circumference (cm) in the EPIC-Potsdam subsample (n = 2,122) with adjustment for sex, age at baseline, educational attainment, occupational activity, sports activity, smoking habits, alcohol intake, energy intake, fat intake, and fruit and vegetable intake.**
(PDF)

Table S3 **Single haplotype analysis on waist circumference (cm) adjusted for body-mass index (kg/m^2^) in the EPIC-Potsdam subsample (n = 2,122) with adjustment for sex, age at baseline, educational attainment, occupational activity, sports activity, smoking habits, alcohol intake, energy intake, fat intake, and fruit and vegetable intake.**
(PDF)

Table S4 **Evaluation of SNP-gender interaction in 2,122 European middle-aged men and women (random population sample).**
(PDF)

Table S5 **Mean p-values of selected SNP patterns in each step of the multi-locus stepwise regression (MSR) with 41 SNPs on Body-mass index (BMI, kg/m^2^), waist circumference (WC, cm) not adjusted and adjusted for BMI in the EPIC-Potsdam subsample (n = 2,122).**
(PDF)

Table S6 **Result of Multi-locus stepwise regression with 41 SNPs on BMI (kg/m^2^) in the EPIC-Potsdam subsample (n = 2,122). Starting with SNP-pairs one SNP at a time was added to the ‘best’ patterns in the interim step. Selection criterion in every step was a decrease of corrected AIC (AICc, lower values are better) and a global p value below a given threshold (2-SNPs: 0.05, 3- and more SNPs: 1/10i-1, where i denote the number of simultaneously analyzed SNPs in each step). SNP numbers correspond to identification number in** **Table 2** **of the main text.**
(PDF)

Table S7 **Result of Multi-locus stepwise regression with 41 SNPs on waist circumference (cm) in the EPIC-Potsdam subsample (n = 2,122). Starting with SNP-pairs one SNP at a time was added to the ‘best’ patterns in the interim step. Selection criterion in every step was a decrease of corrected AIC (AICc, lower values are better) and a global p value below a given threshold (2-SNPs: 0.05, 3- and more SNPs: 1/10?(i−1), where i denote the number of simultaneously analyzed SNPs in each step). SNP numbers correspond to identification number in** **Table 2** **of the main text.**
(PDF)

Table S8 **Result of Multi-locus stepwise regression with 41 SNPs on waist circumference (cm) adjusted for BMI in the EPIC-Potsdam subsample (n = 2,122). Starting with SNP-pairs one SNP at a time was added to the ‘best’ patterns in the interim step. Selection criterion in every step was a decrease of corrected AIC (AICc, lower values are better) and a global p value below a given threshold (2-SNPs: 0.05, 3- and more SNPs: 1/10?(i−1), where i denote the number of simultaneously analyzed SNPs in each step). SNP numbers correspond to identification number in** **Table 2** **of the main text.**
(PDF)

Table S9 **All other associations of the MSR-selected SNP combinations with obesity-related traits (BMI, waist circumference and waist-circumference adjusted for BMI) in 2,122 European middle-aged men and women (random population sample).**
(PDF)

Text S1 **Physiological mechanisms of selected genes in our study.**
(PDF)
