# Supplementary material for: Evaluation of 41 Candidate Gene Variants for Obesity in the EPIC-Potsdam Cohort by Multi-Locus Stepwise Regression
Source: PLoS One. 2013 Jul 12;8(7):e68941. doi: 10.1371/journal.pone.0068941 (PMC3709896; doi:10.1371/journal.pone.0068941)

Figure S1: LD plot for ABCC8 gene of the EPIC Potsdam subsample (2,122).

Disequilibrium coefficient r^2 values were generated using Haploview version 4.2 (Barrett JC et al. Bioinformatics 2005;21(2):263-5). Standard Color Scheme for the LD plots for r^2 were used (for more information see Haploview documentation).

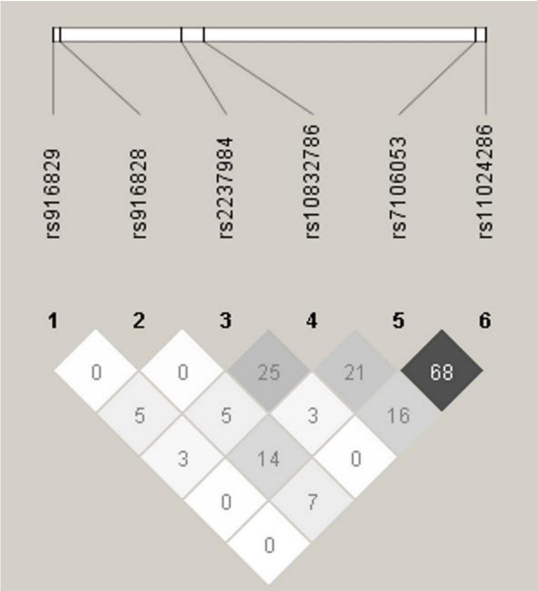

Supplement: Figure S1 — LD plot for ABCC8 gene of the EPIC Potsdam subsample (2,122). Disequilibrium coefficient r2 values were generated using Haploview version 4.2 (Barrett JC et al. Bioinformatics 2005;21(2):263-5). Standard Color Scheme for the LD plots for r2 were used (for more information see Haploview documentation). (PDF) [file pone.0068941.s001.pdf]
