# Supplementary material for: Evaluation of 41 Candidate Gene Variants for Obesity in the EPIC-Potsdam Cohort by Multi-Locus Stepwise Regression
Source: PLoS One. 2013 Jul 12;8(7):e68941. doi: 10.1371/journal.pone.0068941 (PMC3709896; doi:10.1371/journal.pone.0068941)
Supplement: Table S1 — Single haplotype analysis on body-mass index (kg/m2) in the EPIC-Potsdam subsample (n = 2,122) with adjustment for sex, age at baseline, educational attainment, occupational activity, sports activity, smoking habits, alcohol intake, energy intake, fat intake, and fruit and vegetable intake. (PDF) [file pone.0068941.s003.pdf]

Table S1: Single haplotype analysis on body-mass index (kg/m²) in the EPIC-Potsdam subsample (n=2,122) with adjustment for sex, age at baseline, educational attainment, occupational activity, sports activity, smoking habits, alcohol intake, energy intake, fat intake, and fruit and vegetable intake.

Gene	Haplotype	Frequency	Beta	Std.err	p-value
LEPR	221	0.256	-0.16	0.14	0.2614
	112	0.161	-0.11	0.17	0.5106
	111	0.374	0.04	0.13	0.7728
	121	0.199	0.23	0.16	0.1409
HSD11B1	111	0.830	-0.18	0.16	0.2682
	222	0.052	-0.01	0.28	0.9853
	212	0.064	0.23	0.25	0.3642
	211	0.053	0.30	0.28	0.2876
TBC1D1	1111112112111	0.055	-0.46	0.30	0.1295
	2112112112111	0.093	0.10	0.23	0.6557
FABP2	22	0.259	-0.22	0.14	0.1095
	12	0.174	-0.05	0.16	0.7808
	11	0.566	0.20	0.12	0.1038
ABCC8	212111	0.093	-0.43	0.21	0.0460
	121122	0.089	-0.17	0.22	0.4330
	122111	0.078	-0.09	0.23	0.7062
	111111	0.400	0.15	0.12	0.2212
	112222	0.112	0.32	0.19	0.0939
MC4R	11	0.765	-0.34	0.14	0.0154
	22	0.208	0.40	0.15	0.0062

1 = major allele, 2=minor allele
