# Supplementary material for: Evaluation of 41 Candidate Gene Variants for Obesity in the EPIC-Potsdam Cohort by Multi-Locus Stepwise Regression
Source: PLoS One. 2013 Jul 12;8(7):e68941. doi: 10.1371/journal.pone.0068941 (PMC3709896; doi:10.1371/journal.pone.0068941)
Supplement: Table S2 — Single haplotype analysis on waist circumference (cm) in the EPIC-Potsdam subsample (n = 2,122) with adjustment for sex, age at baseline, educational attainment, occupational activity, sports activity, smoking habits, alcohol intake, energy intake, fat intake, and fruit and vegetable intake. (PDF) [file pone.0068941.s004.pdf]

Table S2: Single haplotype analysis on waist circumference (cm) in the EPIC-Potsdam subsample (n=2,122) with adjustment for sex, age at baseline, educational attainment, occupational activity, sports activity, smoking habits, alcohol intake, energy intake, fat intake, and fruit and vegetable intake.

Gene	Haplotype	Frequency	Beta	Std.err	p-value
LEPR	221	0.256	-0.71	0.37	0.0516
	112	0.161	-0.13	0.43	0.7534
	111	0.374	0.29	0.33	0.3751
	121	0.199	0.46	0.40	0.2579
HSD11B1	111	0.830	-0.58	0.42	0.1753
	222	0.052	0.00	0.71	0.9978
	212	0.064	0.70	0.65	0.2816
	211	0.053	0.98	0.72	0.1749
TBC1D1	1111112112111	0.055	-0.66	0.77	0.3960
	2112112112111	0.093	0.49	0.59	0.4123
FABP2	12	0.174	-0.40	0.42	0.3422
	22	0.259	-0.34	0.36	0.3357
	11	0.566	0.49	0.31	0.1213
ABCC8	212111	0.093	-0.61	0.55	0.2662
	122111	0.078	-0.29	0.60	0.6312
	121122	0.089	-0.09	0.57	0.8810
	112222	0.112	0.23	0.49	0.6350
	111111	0.400	0.39	0.32	0.2209
MC4R	11	0.765	-0.90	0.36	0.0130
	22	0.208	1.11	0.38	0.0033

1 = major allele, 2=minor allele
