# Supplementary material for: Evaluation of 41 Candidate Gene Variants for Obesity in the EPIC-Potsdam Cohort by Multi-Locus Stepwise Regression
Source: PLoS One. 2013 Jul 12;8(7):e68941. doi: 10.1371/journal.pone.0068941 (PMC3709896; doi:10.1371/journal.pone.0068941)
Supplement: Table S3 — Single haplotype analysis on waist circumference (cm) adjusted for body-mass index (kg/m2) in the EPIC-Potsdam subsample (n = 2,122) with adjustment for sex, age at baseline, educational attainment, occupational activity, sports activity, smoking habits, alcohol intake, energy intake, fat intake, and fruit and vegetable intake. (PDF) [file pone.0068941.s005.pdf]

Table S3: Single haplotype analysis on waist circumference (cm) adjusted for body-mass index (kg/m²) in the EPIC-Potsdam subsample (n=2,122) with adjustment for sex, age at baseline, educational attainment, occupational activity, sports activity, smoking habits, alcohol intake, energy intake, fat intake, and fruit and vegetable intake.

Gene	Haplotype	Frequency	Beta	Std.err	p-value
LEPR	221	0.256	-0.35	0.17	0.0396
	121	0.199	-0.07	0.19	0.7059
	112	0.161	0.12	0.20	0.5599
	111	0.374	0.21	0.15	0.1720
HSD11B1	111	0.830	-0.16	0.20	0.4192
	222	0.052	0.01	0.33	0.9670
	212	0.064	0.18	0.30	0.5562
	211	0.053	0.30	0.33	0.3711
TBC1D1	2112112112111	0.093	0.25	0.27	0.3583
	1111112112111	0.055	0.38	0.36	0.2830
FABP2	12	0.174	-0.29	0.19	0.1281
	11	0.566	0.03	0.14	0.8177
	22	0.259	0.16	0.17	0.3227
ABCC8	112222	0.112	-0.50	0.23	0.0285
	122111	0.078	-0.09	0.28	0.7526
	111111	0.400	0.04	0.15	0.7634
	121122	0.089	0.31	0.27	0.2375
	212111	0.093	0.36	0.26	0.1543
MC4R	11	0.765	-0.12	0.17	0.4673
	22	0.208	0.19	0.17	0.2682

1 = major allele, 2=minor allele
