# Supplementary material for: Evaluation of 41 Candidate Gene Variants for Obesity in the EPIC-Potsdam Cohort by Multi-Locus Stepwise Regression
Source: PLoS One. 2013 Jul 12;8(7):e68941. doi: 10.1371/journal.pone.0068941 (PMC3709896; doi:10.1371/journal.pone.0068941)
Supplement: Table S4 — Evaluation of SNP-gender interaction in 2,122 European middle-aged men and women (random population sample). (PDF) [file pone.0068941.s006.pdf]

Table S4: Evaluation of SNP-gender interaction in 2,122 European middle-aged men and women (random population sample).

ID	SNP	Gene	BMI (kg/m2)				Waist circumference (cm)				Waist circumference (cm) adjusted for BMI			
			Beta (se) Men	Beta (se) Women	p (Interaction)	Beta difference	Beta (se) Men	Beta (se) Women	p (Interaction)	Beta difference	Beta (se) Men	Beta (se) Women	p (Interaction)	Beta difference
1	rs1137100	LEPR	-0.21 (0.18)	-0.18 (0.19)	0.90	-0.03	-0.87 (0.52)	-0.54 (0.47)	0.81	-0.33	-0.36 (0.25)	-0.15 (0.22)	0.81	-0.21
2	rs1137101	LEPR	0.00 (0.17)	0.02 (0.17)	0.99	-0.02	-0.61 (0.47)	-0.06 (0.41)	0.44	-0.55	-0.60 (0.22)	-0.10 (0.19)	0.44	-0.51
3	rs8179183	LEPR	-0.06 (0.22)	-0.14 (0.22)	0.68	0.08	0.33 (0.63)	-0.44 (0.56)	0.31	0.77	0.48 (0.29)	-0.12 (0.26)	0.31	0.60
4	rs4844880	HSD11B1	0.16 (0.23)	0.18 (0.23)	0.78	-0.02	0.62 (0.65)	0.51 (0.56)	0.67	0.10	0.23 (0.30)	0.12 (0.26)	0.67	0.10
5	rs846910	HSD11B1	0.18 (0.38)	-0.36 (0.37)	0.31	0.54	0.36 (1.06)	-0.96 (0.93)	0.32	1.32	-0.08 (0.49)	-0.16 (0.43)	0.32	0.08
6	rs3753519	HSD11B1	0.16 (0.27)	0.02 (0.26)	0.50	0.15	0.47 (0.76)	0.12 (0.65)	0.51	0.36	0.06 (0.36)	0.08 (0.30)	0.51	-0.01
7	rs11127485	TMEM18	0.10 (0.22)	-0.16 (0.23)	0.44	0.26	-0.04 (0.63)	-0.43 (0.57)	0.72	0.39	-0.28 (0.29)	-0.08 (0.26)	0.72	-0.20
8	rs2241883	FABP1	-0.01 (0.17)	-0.18 (0.17)	0.54	0.17	-0.33 (0.48)	-0.39 (0.42)	0.94	0.06	-0.31 (0.23)	0.00 (0.19)	0.94	-0.31
9	rs7566605	INSIG2	-0.02 (0.18)	-0.10 (0.18)	0.77	0.08	-0.04 (0.50)	0.00 (0.45)	0.90	-0.04	0.00 (0.23)	0.23 (0.21)	0.90	-0.22
10	rs3762521	ALPI	0.07 (0.18)	-0.05 (0.18)	0.77	0.12	0.00 (0.51)	-0.07 (0.45)	0.92	0.07	-0.17 (0.24)	0.04 (0.21)	0.92	-0.21
11	rs1801282	PPARG	-0.09 (0.23)	0.47 (0.23)	0.11	-0.55	-0.44 (0.65)	1.45 (0.56)	0.03	-1.89	-0.22 (0.31)	0.41 (0.26)	0.03	-0.64
12	rs2279027	TBC1D1	0.11 (0.17)	0.07 (0.17)	0.95	0.05	0.43 (0.47)	0.06 (0.42)	0.69	0.37	0.16 (0.22)	-0.08 (0.19)	0.69	0.24
13	rs35859249	TBC1D1	0.39 (0.32)	-0.44 (0.31)	0.16	0.83	1.04 (0.89)	-1.07 (0.76)	0.15	2.10	0.07 (0.42)	-0.09 (0.35)	0.15	0.16
14	rs4832743	TBC1D1	0.06 (0.18)	-0.04 (0.17)	0.87	0.10	-0.32 (0.50)	-0.15 (0.42)	0.57	-0.17	-0.46 (0.23)	-0.06 (0.19)	0.57	-0.40
15	rs10517456	TBC1D1	0.00 (0.17)	-0.06 (0.17)	0.73	0.06	0.13 (0.47)	0.24 (0.42)	0.95	-0.11	0.12 (0.22)	0.37 (0.19)	0.95	-0.25
16	rs9999507	TBC1D1	0.26 (0.17)	-0.18 (0.16)	0.09	0.44	0.25 (0.47)	-0.50 (0.41)	0.30	0.75	-0.39 (0.22)	-0.10 (0.19)	0.30	-0.29
17	rs6845120	TBC1D1	0.32 (0.18)	-0.12 (0.18)	0.11	0.44	0.39 (0.51)	-0.44 (0.46)	0.26	0.83	-0.40 (0.24)	-0.16 (0.21)	0.26	-0.24
18	rs6823014	TBC1D1	-0.33 (0.17)	-0.07 (0.17)	0.29	-0.27	-0.35 (0.47)	-0.09 (0.42)	0.67	-0.26	0.47 (0.22)	0.05 (0.19)	0.67	0.42
19	rs10009706	TBC1D1	0.31 (0.19)	-0.15 (0.19)	0.12	0.45	0.40 (0.52)	-0.37 (0.48)	0.30	0.77	-0.37 (0.24)	-0.04 (0.22)	0.30	-0.33
20	rs2303422	TBC1D1	0.30 (0.23)	-0.05 (0.23)	0.21	0.35	0.51 (0.65)	-0.44 (0.57)	0.19	0.95	-0.23 (0.30)	-0.32 (0.26)	0.19	0.09
21	rs1344603	TBC1D1	-0.04 (0.18)	0.06 (0.18)	0.65	-0.10	0.26 (0.51)	0.15 (0.44)	0.87	0.12	0.37 (0.24)	0.02 (0.20)	0.87	0.35
22	rs637797	TBC1D1	0.07 (0.18)	-0.52 (0.18)	0.04	0.59	-0.06 (0.51)	-1.18 (0.45)	0.16	1.13	-0.22 (0.24)	-0.04 (0.21)	0.16	-0.19
23	rs6837834	TBC1D1	0.11 (0.24)	0.19 (0.26)	0.97	-0.08	-0.10 (0.68)	0.52 (0.65)	0.66	-0.63	-0.38 (0.32)	0.10 (0.30)	0.66	-0.48
24	rs13110318	TBC1D1	-0.27 (0.30)	-0.11 (0.31)	0.56	-0.16	-0.48 (0.86)	-0.45 (0.77)	0.73	-0.03	0.19 (0.40)	-0.20 (0.35)	0.73	0.40
25	rs3816873	MTTP	-0.13 (0.19)	-0.02 (0.19)	0.81	-0.11	-0.26 (0.53)	-0.01 (0.47)	0.84	-0.25	0.07 (0.25)	0.04 (0.22)	0.84	0.03
26	rs1799883	FABP2	-0.10 (0.19)	-0.34 (0.19)	0.29	0.24	0.07 (0.53)	-0.70 (0.48)	0.22	0.76	0.32 (0.25)	0.06 (0.22)	0.22	0.26
27	rs6857641	FABP2	-0.06 (0.16)	-0.26 (0.17)	0.42	0.20	-0.05 (0.46)	-0.69 (0.42)	0.35	0.64	0.11 (0.21)	-0.11 (0.19)	0.35	0.22
28	rs13283456	PTGES2	-0.02 (0.22)	0.24 (0.22)	0.32	-0.26	0.13 (0.61)	0.55 (0.55)	0.52	-0.42	0.18 (0.28)	0.01 (0.25)	0.52	0.17
29	rs7903146	TCF7L2	-0.09 (0.18)	-0.17 (0.19)	0.86	0.08	-0.06 (0.52)	-0.63 (0.48)	0.40	0.57	0.17 (0.24)	-0.25 (0.22)	0.40	0.42
30	rs916829	ABCC8	-0.12 (0.22)	-0.33 (0.24)	0.62	0.22	-0.35 (0.62)	-0.40 (0.59)	0.95	0.05	-0.05 (0.29)	0.34 (0.27)	0.95	-0.39
31	rs916828	ABCC8	-0.02 (0.19)	-0.12 (0.19)	0.97	0.10	0.25 (0.54)	-0.16 (0.47)	0.81	0.41	0.30 (0.25)	0.10 (0.21)	0.81	0.20
32	rs2237984	ABCC8	-0.17 (0.17)	0.03 (0.17)	0.37	-0.20	-0.66 (0.47)	-0.11 (0.42)	0.36	-0.56	-0.23 (0.22)	-0.17 (0.19)	0.36	-0.07
33	rs10832786	ABCC8	0.08 (0.23)	0.35 (0.24)	0.52	-0.26	-0.18 (0.65)	0.26 (0.59)	0.70	-0.45	-0.39 (0.31)	-0.50 (0.27)	0.70	0.11
34	rs7106053	ABCC8	0.11 (0.18)	-0.02 (0.17)	0.65	0.12	0.26 (0.50)	-0.25 (0.43)	0.46	0.51	0.00 (0.23)	-0.21 (0.20)	0.46	0.21
35	rs11024286	ABCC8	0.03 (0.18)	-0.05 (0.18)	0.78	0.08	0.24 (0.52)	-0.37 (0.44)	0.39	0.60	0.16 (0.24)	-0.25 (0.20)	0.39	0.41
36	rs1520220	IGF1	0.44 (0.21)	0.21 (0.22)	0.43	0.23	0.86 (0.60)	0.74 (0.53)	0.80	0.12	-0.23 (0.28)	0.28 (0.24)	0.80	-0.51
37	rs9939609	FTO	0.56 (0.17)	0.14 (0.17)	0.12	0.41	1.55 (0.48)	0.08 (0.43)	0.03	1.46	0.16 (0.23)	-0.24 (0.20)	0.03	0.40
38	rs2297508	SREBF1	0.19 (0.17)	0.13 (0.17)	0.66	0.06	0.58 (0.49)	0.23 (0.43)	0.50	0.35	0.11 (0.23)	-0.06 (0.19)	0.50	0.17
39	rs1805081	NPC1	-0.05 (0.17)	-0.02 (0.17)	0.99	-0.03	-0.32 (0.48)	-0.08 (0.42)	0.83	-0.24	-0.20 (0.23)	-0.04 (0.19)	0.83	-0.17
40	rs17700144	MC4R	0.17 (0.20)	0.60 (0.20)	0.12	-0.43	0.77 (0.57)	1.44 (0.50)	0.34	-0.66	0.36 (0.27)	0.11 (0.23)	0.34	0.24
41	rs10871777	MC4R	0.13 (0.19)	0.51 (0.19)	0.16	-0.38	0.68 (0.54)	1.16 (0.48)	0.49	-0.48	0.37 (0.25)	0.04 (0.22)	0.49	0.33

Multiple linear regression model for body mass index (BMI) and waist circumference (WC) based on adjustment for sex, age, education, occupational activity smoking, alcohol intake, energy intake, fat intake, and fruit and vegetable intake.
SNPs are coded as 0, 1, 2 for minor allele count.
