# Supplementary material for: Evaluation of 41 Candidate Gene Variants for Obesity in the EPIC-Potsdam Cohort by Multi-Locus Stepwise Regression
Source: PLoS One. 2013 Jul 12;8(7):e68941. doi: 10.1371/journal.pone.0068941 (PMC3709896; doi:10.1371/journal.pone.0068941)
Supplement: Table S5 — Mean p-values of selected SNP patterns in each step of the multi-locus stepwise regression (MSR) with 41 SNPs on Body-mass index (BMI, kg/m2), waist circumference (WC, cm) not adjusted and adjusted for BMI in the EPIC-Potsdam subsample (n = 2,122). (PDF) [file pone.0068941.s007.pdf]

Table S5: Mean p-values of selected SNP patterns in each step of the multi-locus stepwise regression (MSR) with 41 SNPs on Body-mass index (BMI, kg/m²), waist circumference (WC, cm) not adjusted and adjusted for BMI in the EPIC-Potsdam subsample (n=2,122).

SNPs at a time	BMI	WC	WC adj. BMI
2	2.62E-02	2.34E-02	2.82E-02
3	4.62E-03	4.37E-03	7.09E-03
4	5.43E-04	5.67E-04	-
5	6.49E-05	3.94E-05	-
6	7.71E-06	8.78E-06	-
