# Supplementary material for: Evaluation of 41 Candidate Gene Variants for Obesity in the EPIC-Potsdam Cohort by Multi-Locus Stepwise Regression
Source: PLoS One. 2013 Jul 12;8(7):e68941. doi: 10.1371/journal.pone.0068941 (PMC3709896; doi:10.1371/journal.pone.0068941)
Supplement: Table S6 — Result of Multi-locus stepwise regression with 41 SNPs on BMI (kg/m2) in the EPIC-Potsdam subsample (n = 2,122). Starting with SNP-pairs one SNP at a time was added to the ‘best’ patterns in the interim step. Selection criterion in every step was a decrease of corrected AIC (AICc, lower values are better) and a global p value below a given threshold (2-SNPs: 0.05, 3- and more SNPs: 1/10i-1, where i denote the number of simultaneously analyzed SNPs in each step). SNP numbers correspond to identification number in Table 2 of the main text. (PDF) [file pone.0068941.s008.pdf]

Table S6: Result of Multi-locus stepwise regression with 41 SNPs on BMI (kg/m²) in the EPIC-Potsdam subsample (n=2,122). Starting with SNP-pairs one SNP at a time was added to the 'best' patterns in the interim step. Selection criterion in every step was a decrease of corrected AIC (AICc, lower values are better) and a global p value below a given threshold (2-SNPs: 0.05, 3- and more SNPs: 1/10i-1, where i denote the number of simultaneously analyzed SNPs in each step). SNP numbers correspond to identification number in Table 2 of the main text.

SNP 1	SNP 2	SNP 3	SNP 4	SNP 5	SNP 6	Global p-value	AICc
**2 SNPs at a time**							
33	40					9.10E-04	11847.53
22	40					1.78E-03	11848.52
37	40					2.48E-03	11849.23
15	18					3.02E-03	11849.66
33	41					4.59E-03	11850.80
37	41					4.61E-03	11850.57
22	41					5.19E-03	11850.83
40	41					6.16E-03	11852.06
29	40					6.65E-03	11851.55
18	40					7.46E-03	11851.62
26	37					7.72E-03	11851.69
11	40					8.31E-03	11852.00
30	31					8.78E-03	11851.97
30	40					9.46E-03	11852.26
27	40					9.67E-03	11852.18
26	40					9.70E-03	11852.19
18	41					9.77E-03	11852.21
22	27					1.06E-02	11852.38
39	40					1.06E-02	11852.39
22	37					1.13E-02	11852.53
24	40					1.14E-02	11852.63
20	22					1.28E-02	11852.87
13	40					1.33E-02	11852.95
22	33					1.36E-02	11852.99
38	40					1.40E-02	11852.99
34	40					1.48E-02	11853.11
23	40					1.49E-02	11853.19
19	41					1.51E-02	11853.16
6	40					1.56E-02	11853.27
11	22					1.57E-02	11853.29
5	40					1.63E-02	11853.36
6	37					1.67E-02	11853.42
22	26					1.74E-02	11853.48
4	22					1.96E-02	11853.73
29	41					1.96E-02	11853.74
19	40					2.03E-02	11853.82
32	40					2.04E-02	11853.83
1	40					2.04E-02	11853.83
22	36					2.16E-02	11853.95
18	22					2.25E-02	11854.04
30	41					2.31E-02	11854.06
11	41					2.31E-02	11854.06
4	40					2.33E-02	11854.09
14	40					2.36E-02	11854.15
15	40					2.42E-02	11854.21

SNP 1	SNP 2	SNP 3	SNP 4	SNP 5	SNP 6	Global p-value	AICc
27	37					2.51E-02	11854.28
25	40					2.57E-02	11854.34
3	40					2.59E-02	11854.30
15	36					2.60E-02	11854.36
8	40					2.60E-02	11854.37
1	36					2.75E-02	11854.42
22	38					2.90E-02	11854.61
18	37					2.90E-02	11854.61
26	41					2.96E-02	11854.65
24	41					3.08E-02	11854.65
17	41					3.29E-02	11854.89
27	41					3.31E-02	11854.90
21	22					3.41E-02	11854.85
3	22					3.43E-02	11854.87
36	37					3.46E-02	11855.00
17	22					3.52E-02	11855.03
24	37					3.79E-02	11855.07
22	29					3.87E-02	11855.25
6	41					3.97E-02	11855.16
22	39					3.97E-02	11855.31
17	40					4.04E-02	11855.34
28	40					4.06E-02	11855.21
13	41					4.08E-02	11855.22
38	41					4.17E-02	11855.42
21	40					4.17E-02	11855.42
30	37					4.18E-02	11855.42
29	37					4.19E-02	11855.43
8	22					4.28E-02	11855.48
2	40					4.31E-02	11855.49
26	36					4.35E-02	11855.34
1	22					4.37E-02	11855.52
31	40					4.39E-02	11855.53
22	23					4.40E-02	11855.37
16	40					4.41E-02	11855.55
12	40					4.44E-02	11855.56
12	14					4.46E-02	11855.57
4	37					4.51E-02	11855.59
22	28					4.63E-02	11855.65
5	37					4.70E-02	11855.50
9	40					4.75E-02	11855.71
22	24					4.78E-02	11855.54
4	41					4.78E-02	11855.54
7	40					4.81E-02	11855.55
5	41					4.88E-02	11855.58
7	29					4.98E-02	11855.62
35	40					4.99E-02	11855.82
**3 SNPs at a time**							
15	18	40				1.94E-04	11843.25
15	18	36				3.84E-04	11844.85
15	18	22				7.60E-04	11846.63
22	33	40				8.24E-04	11846.62
22	26	40				8.90E-04	11846.80
26	37	40				9.15E-04	11846.93
22	27	40				1.01E-03	11847.18

SNP 1	SNP 2	SNP 3	SNP 4	SNP 5	SNP 6	Global p-value	AICc
15	18	41				1.02E-03	11847.36
15	33	40				1.05E-03	11847.19
22	37	40				1.33E-03	11847.84
26	36	37				1.40E-03	11847.85
26	37	41				1.78E-03	11848.54
27	37	40				1.80E-03	11848.58
6	7	29				2.11E-03	11848.78
37	40	41				2.13E-03	11848.90
8	15	36				2.17E-03	11849.04
15	18	37				2.38E-03	11849.49
30	37	41				2.42E-03	11849.15
29	37	40				2.50E-03	11849.22
15	18	30				2.55E-03	11849.21
13	15	18				2.66E-03	11849.31
30	31	40				2.84E-03	11849.46
22	37	41				2.90E-03	11849.75
15	17	22				3.10E-03	11849.92
22	33	41				3.23E-03	11849.83
26	38	40				3.28E-03	11850.05
9	36	40				3.58E-03	11850.07
11	22	27				3.84E-03	11850.24
15	36	37				4.00E-03	11850.34
7	29	40				4.02E-03	11850.25
23	30	31				4.08E-03	11850.28
22	26	41				4.47E-03	11850.61
18	30	31				4.61E-03	11850.68
34	35	40				4.64E-03	11850.57
6	29	41				4.66E-03	11850.71
1	27	40				4.81E-03	11851.01
30	31	36				4.96E-03	11850.73
4	7	29				5.01E-03	11850.75
15	16	36				5.16E-03	11851.18
15	39	40				5.27E-03	11851.23
20	26	37				5.28E-03	11851.01
21	22	35				5.43E-03	11851.07
18	27	40				5.62E-03	11851.39
15	19	40				5.80E-03	11851.47
27	36	37				5.97E-03	11851.31
7	11	40				5.98E-03	11851.16
20	22	39				6.20E-03	11851.40
15	19	36				6.30E-03	11851.43
18	40	41				6.43E-03	11851.30
30	31	33				7.23E-03	11851.59
4	7	40				7.33E-03	11851.63
4	30	31				7.58E-03	11851.70
20	22	37				7.60E-03	11851.89
15	19	41				8.06E-03	11852.31
5	30	31				8.26E-03	11851.90
13	39	40				8.33E-03	11851.92
28	36	40				8.37E-03	11851.93
7	30	31				8.42E-03	11851.95
4	11	40				8.58E-03	11851.99
15	16	40				8.76E-03	11852.52
7	31	40				9.20E-03	11852.15

SNP 1	SNP 2	SNP 3	SNP 4	SNP 5	SNP 6	Global p-value	AICc
12	14	22				9.57E-03	11852.75
12	14	25				9.75E-03	11852.79
13	36	40				9.77E-03	11852.29
24	39	40				9.91E-03	11852.33
**4 SNPs at a time**							
26	36	37	40			9.01E-05	11841.45
4	7	11	40			1.05E-04	11841.88
22	27	33	40			1.85E-04	11843.13
27	36	37	40			1.93E-04	11843.23
15	18	36	41			2.27E-04	11843.65
22	33	37	40			2.30E-04	11843.64
13	15	18	36			2.35E-04	11843.69
15	18	35	36			2.59E-04	11844.11
27	37	38	40			2.60E-04	11844.12
20	26	36	37			2.95E-04	11844.23
14	22	37	40			3.01E-04	11844.49
4	27	37	40			3.01E-04	11844.30
11	22	27	40			3.05E-04	11844.38
11	22	33	40			3.46E-04	11844.62
22	27	37	40			3.49E-04	11844.86
24	33	39	40			3.49E-04	11844.64
15	18	29	41			3.63E-04	11844.81
14	27	37	40			3.78E-04	11845.06
26	37	39	40			3.79E-04	11844.91
20	22	39	40			3.81E-04	11844.92
20	26	37	40			3.94E-04	11845.01
6	7	29	40			4.13E-04	11845.02
6	26	37	40			4.15E-04	11845.04
27	33	37	40			4.25E-04	11845.09
22	33	39	40			4.46E-04	11845.31
13	15	18	41			4.59E-04	11845.28
26	36	37	41			4.62E-04	11845.40
12	14	22	36			4.65E-04	11845.41
6	26	37	41			5.19E-04	11845.57
4	7	29	40			5.20E-04	11845.55
6	27	37	40			5.40E-04	11845.64
18	26	37	40			5.66E-04	11845.78
15	26	37	40			5.77E-04	11845.83
27	36	37	41			6.07E-04	11845.95
6	15	18	41			6.11E-04	11845.96
26	33	37	40			6.13E-04	11846.09
22	33	37	41			6.31E-04	11846.04
21	22	33	35			6.41E-04	11846.08
4	22	27	40			6.44E-04	11846.21
18	26	36	37			6.48E-04	11846.11
23	26	37	40			6.53E-04	11846.12
11	26	37	40			6.66E-04	11846.17
30	31	33	40			6.86E-04	11846.19
17	26	37	41			6.91E-04	11846.26
27	32	37	40			7.16E-04	11846.48
8	22	33	40			7.17E-04	11846.35
11	27	37	40			7.25E-04	11846.32
14	22	37	41			7.45E-04	11846.77
5	26	37	40			7.91E-04	11846.58

SNP 1	SNP 2	SNP 3	SNP 4	SNP 5	SNP 6	Global p-value	AICc
16	26	37	40			7.94E-04	11846.59
7	30	31	40			8.20E-04	11846.61
2	26	37	40			8.20E-04	11846.67
13	15	18	29			8.30E-04	11846.84
12	22	37	40			8.49E-04	11847.11
1	27	33	40			8.85E-04	11846.85
13	15	18	30			8.92E-04	11846.84
26	29	37	41			9.20E-04	11847.10
16	22	37	40			9.33E-04	11847.13
6	26	36	37			9.40E-04	11847.00
11	15	33	40			9.62E-04	11847.06
6	30	37	41			9.83E-04	11847.03
**5 SNPs at a time**							
11	26	33	37	40		3.45E-05	11839.67
13	15	18	29	31		3.68E-05	11839.37
4	27	36	37	40		3.71E-05	11839.83
11	23	26	37	40		3.76E-05	11839.86
11	22	33	37	40		5.18E-05	11840.30
4	22	27	33	40		5.81E-05	11840.55
7	13	15	18	29		5.86E-05	11840.44
23	26	33	37	40		6.32E-05	11840.74
1	13	15	18	29		6.50E-05	11840.68
6	26	36	37	40		6.62E-05	11840.73
6	27	36	37	40		6.86E-05	11841.17
17	26	36	37	40		7.07E-05	11840.88
26	33	36	37	40		7.10E-05	11841.01
4	27	33	37	40		7.13E-05	11841.26
4	26	36	37	41		7.35E-05	11841.08
6	26	36	37	41		7.56E-05	11841.03
4	26	36	37	40		7.65E-05	11841.18
23	26	36	37	40		8.60E-05	11841.34
3	15	18	35	36		9.58E-05	11841.58
6	11	26	37	40		9.91E-05	11841.98
**6 SNPs at a time**							
4	19	26	36	37	40	3.45E-06	11834.69
7	13	15	18	29	30	6.82E-06	11836.16
7	13	15	18	29	31	7.53E-06	11836.37
4	19	26	36	37	41	7.53E-06	11836.37
23	26	33	36	37	40	8.18E-06	11836.55
1	7	13	15	18	29	8.21E-06	11836.56
11	23	26	33	37	40	8.50E-06	11836.63
4	17	26	36	37	40	9.47E-06	11836.49
1	13	15	18	29	38	9.70E-06	11836.31
