# Supplementary material for: Evaluation of 41 Candidate Gene Variants for Obesity in the EPIC-Potsdam Cohort by Multi-Locus Stepwise Regression
Source: PLoS One. 2013 Jul 12;8(7):e68941. doi: 10.1371/journal.pone.0068941 (PMC3709896; doi:10.1371/journal.pone.0068941)
Supplement: Table S7 — Result of Multi-locus stepwise regression with 41 SNPs on waist circumference (cm) in the EPIC-Potsdam subsample (n = 2,122). Starting with SNP-pairs one SNP at a time was added to the ‘best’ patterns in the interim step. Selection criterion in every step was a decrease of corrected AIC (AICc, lower values are better) and a global p value below a given threshold (2-SNPs: 0.05, 3- and more SNPs: 1/10?(i−1), where i denote the number of simultaneously analyzed SNPs in each step). SNP numbers correspond to identification number in Table 2 of the main text. (PDF) [file pone.0068941.s009.pdf]

Table S7: Result of Multi-locus stepwise regression with 41 SNPs on waist circumference (cm) in the EPIC-Potsdam subsample (n=2,122). Starting with SNP-pairs one SNP at a time was added to the 'best' patterns in the interim step. Selection criterion in every step was a decrease of corrected AIC (AICc, lower values are better) and a global p value below a given threshold (2-SNPs: 0.05, 3- and more SNPs: 1/10^(i-1), where i denote the number of simultaneously analyzed SNPs in each step). SNP numbers correspond to identification number in Table 2 of the main text.

SNP 1	SNP 2	SNP 3	SNP 4	SNP 5	SNP 6	Global p-value	AICc
**2 SNPs at a time**							
40	41					3.27E-03	15868.95
1	40					3.62E-03	15868.09
22	41					3.72E-03	15868.15
37	40					4.77E-03	15868.69
11	40					4.81E-03	15868.94
33	40					4.99E-03	15869.01
19	41					5.44E-03	15868.98
19	40					5.59E-03	15869.04
26	37					5.84E-03	15869.13
18	40					6.60E-03	15869.40
24	40					6.62E-03	15869.59
23	40					6.95E-03	15869.68
29	40					7.15E-03	15869.74
4	40					7.69E-03	15869.89
5	40					7.81E-03	15869.92
18	41					8.04E-03	15869.83
17	41					8.93E-03	15870.05
39	40					9.91E-03	15870.28
4	22					9.95E-03	15870.41
30	40					1.01E-02	15870.45
27	40					1.02E-02	15870.34
11	22					1.03E-02	15870.49
13	40					1.04E-02	15870.49
22	36					1.05E-02	15870.40
6	40					1.06E-02	15870.53
1	41					1.07E-02	15870.45
17	40					1.21E-02	15870.72
8	40					1.24E-02	15870.77
15	18					1.33E-02	15870.92
38	40					1.38E-02	15871.00
2	40					1.40E-02	15871.04
21	36					1.45E-02	15871.11
34	40					1.46E-02	15871.13
4	41					1.47E-02	15871.20
32	40					1.52E-02	15871.22
37	41					1.62E-02	15871.36
33	41					1.72E-02	15871.52
30	31					1.93E-02	15871.75
11	41					1.94E-02	15871.76
1	36					1.96E-02	15871.78
26	40					2.01E-02	15871.84
1	22					2.01E-02	15871.84
25	40					2.03E-02	15871.86

SNP 1	SNP 2	SNP 3	SNP 4	SNP 5	SNP 6	Global p-value	AICc
20	22					2.03E-02	15871.85
8	22					2.06E-02	15871.89
24	41					2.06E-02	15871.88
22	37					2.29E-02	15872.12
14	40					2.32E-02	15872.15
21	40					2.41E-02	15872.24
7	40					2.45E-02	15872.23
16	40					2.51E-02	15872.33
15	40					2.54E-02	15872.35
5	41					2.72E-02	15872.44
6	41					2.76E-02	15872.47
35	40					2.81E-02	15872.58
12	40					2.82E-02	15872.58
16	36					2.85E-02	15872.61
22	27					2.87E-02	15872.63
27	37					2.93E-02	15872.67
6	22					3.16E-02	15872.74
3	40					3.23E-02	15872.79
2	41					3.25E-02	15872.91
29	41					3.27E-02	15872.91
31	40					3.30E-02	15872.94
9	40					3.45E-02	15873.04
13	41					3.47E-02	15872.93
10	40					3.50E-02	15873.07
27	41					3.50E-02	15873.07
22	39					3.69E-02	15873.19
39	41					3.79E-02	15873.25
38	41					3.82E-02	15873.26
7	29					4.01E-02	15873.22
30	41					4.04E-02	15873.24
22	33					4.06E-02	15873.25
27	36					4.09E-02	15873.42
22	28					4.11E-02	15873.43
22	29					4.11E-02	15873.43
21	22					4.15E-02	15873.29
22	23					4.27E-02	15873.35
22	24					4.31E-02	15873.37
22	38					4.39E-02	15873.58
14	22					4.45E-02	15873.60
5	22					4.45E-02	15873.44
34	41					4.63E-02	15873.70
3	22					4.64E-02	15873.52
1	11					4.67E-02	15873.53
8	41					4.69E-02	15873.73
8	24					4.80E-02	15873.59
23	41					4.81E-02	15873.59
15	22					4.97E-02	15873.85
**3 SNPs at a time**							
27	32	37				5.26E-04	15863.76
15	18	40				6.51E-04	15864.16
26	37	40				1.10E-03	15865.42
1	27	40				1.60E-03	15866.34
7	29	40				1.93E-03	15866.63

SNP 1	SNP 2	SNP 3	SNP 4	SNP 5	SNP 6	Global p-value	AICc
1	23	40				1.98E-03	15866.68
26	36	37				2.00E-03	15866.74
15	18	41				2.02E-03	15867.11
26	37	41				2.62E-03	15867.54
4	11	40				2.72E-03	15867.40
15	18	36				2.81E-03	15867.71
15	18	22				2.87E-03	15868.01
19	40	41				3.07E-03	15867.74
1	40	41				3.10E-03	15867.76
18	30	31				3.15E-03	15867.82
4	7	40				3.22E-03	15867.79
1	22	41				3.23E-03	15868.06
1	11	40				3.27E-03	15867.82
27	32	36				3.32E-03	15867.94
21	36	40				3.40E-03	15868.00
18	40	41				3.45E-03	15867.99
37	39	40				3.78E-03	15868.25
15	33	40				3.78E-03	15868.25
7	11	40				3.85E-03	15868.19
18	23	40				3.98E-03	15868.27
19	25	40				4.18E-03	15868.49
21	36	41				4.41E-03	15868.62
19	24	41				4.47E-03	15868.53
5	18	40				4.64E-03	15868.62
37	40	41				4.69E-03	15868.65
1	27	41				4.74E-03	15869.01
11	36	40				4.98E-03	15868.78
4	36	40				5.33E-03	15868.93
5	18	41				5.34E-03	15868.94
4	28	40				6.83E-03	15869.51
9	36	40				6.99E-03	15869.73
30	31	40				7.21E-03	15869.63
21	22	36				7.24E-03	15869.81
27	36	37				7.33E-03	15869.85
15	18	30				7.35E-03	15869.68
5	17	41				7.39E-03	15869.69
5	17	40				7.51E-03	15869.73
6	7	29				7.90E-03	15869.84
3	7	40				7.91E-03	15869.85
13	36	40				8.28E-03	15869.95
16	27	41				9.03E-03	15870.99
**4 SNPs at a time**							
27	32	36	37			4.05E-05	15857.59
27	32	37	40			8.03E-05	15859.20
4	7	11	40			8.28E-05	15859.40
26	36	37	40			1.49E-04	15860.66
23	27	32	37			1.84E-04	15861.30
27	32	36	40			3.62E-04	15862.76
27	32	37	41			3.79E-04	15862.95
27	36	37	40			3.88E-04	15862.92
26	37	39	40			3.98E-04	15863.07
20	26	37	40			4.05E-04	15863.12
6	7	29	40			4.28E-04	15863.14

SNP 1	SNP 2	SNP 3	SNP 4	SNP 5	SNP 6	Global p-value	AICc
4	22	28	40			4.44E-04	15863.23
4	28	36	40			5.23E-04	15863.61
15	18	36	41			5.34E-04	15863.66
4	7	29	40			5.39E-04	15863.68
11	26	37	40			5.47E-04	15863.74
16	26	37	40			6.28E-04	15864.07
10	26	37	40			6.58E-04	15864.31
1	27	33	40			6.61E-04	15864.20
5	26	37	40			6.80E-04	15864.26
26	36	37	41			7.56E-04	15864.66
1	11	22	40			8.40E-04	15864.77
20	26	36	37			9.24E-04	15865.00
1	26	37	40			9.43E-04	15865.20
15	18	29	41			9.53E-04	15865.23
4	11	36	40			9.54E-04	15865.00
9	36	38	40			9.74E-04	15865.13
15	26	37	40			9.84E-04	15865.15
6	26	37	40			9.95E-04	15865.18
**5 SNPs at a time**							
27	32	36	37	40		1.14E-05	15854.59
4	27	32	36	40		2.55E-05	15857.06
26	32	36	37	40		3.10E-05	15857.19
23	27	32	36	37		3.25E-05	15857.06
4	27	32	37	40		4.79E-05	15857.97
4	27	36	37	40		5.84E-05	15858.86
23	27	32	37	40		6.90E-05	15858.84
**6 SNPs at a time**							
23	27	32	36	37	40	7.80E-06	15853.71
4	27	32	36	37	40	9.76E-06	15854.23
