# Supplementary material for: Evaluation of 41 Candidate Gene Variants for Obesity in the EPIC-Potsdam Cohort by Multi-Locus Stepwise Regression
Source: PLoS One. 2013 Jul 12;8(7):e68941. doi: 10.1371/journal.pone.0068941 (PMC3709896; doi:10.1371/journal.pone.0068941)
Supplement: Table S8 — Result of Multi-locus stepwise regression with 41 SNPs on waist circumference (cm) adjusted for BMI in the EPIC-Potsdam subsample (n = 2,122). Starting with SNP-pairs one SNP at a time was added to the ‘best’ patterns in the interim step. Selection criterion in every step was a decrease of corrected AIC (AICc, lower values are better) and a global p value below a given threshold (2-SNPs: 0.05, 3- and more SNPs: 1/10?(i−1), where i denote the number of simultaneously analyzed SNPs in each step). SNP numbers correspond to identification number in Table 2 of the main text. (PDF) [file pone.0068941.s010.pdf]

Table S8: Result of Multi-locus stepwise regression with 41 SNPs on waist circumference (cm) adjusted for BMI in the EPIC-Potsdam subsample (n=2,122). Starting with SNP-pairs one SNP at a time was added to the 'best' patterns in the interim step. Selection criterion in every step was a decrease of corrected AIC (AICc, lower values are better) and a global p value below a given threshold (2-SNPs: 0.05, 3- and more SNPs: 1/10^(i-1), where i denote the number of simultaneously analyzed SNPs in each step). SNP numbers correspond to identification number in Table 2 of the main text.

SNP 1	SNP 2	SNP 3	Global p-value	AICc
**2 SNPs at a time**				
26	33		2.52E-03	12591.85
1	33		8.38E-03	12594.28
27	33		9.97E-03	12594.51
15	33		1.30E-02	12595.10
20	33		1.46E-02	12595.41
3	33		2.14E-02	12596.17
16	33		2.64E-02	12596.66
2	33		2.94E-02	12596.90
4	33		3.00E-02	12596.86
33	40		3.03E-02	12596.88
21	33		3.44E-02	12597.14
16	30		3.54E-02	12597.31
1	39		3.62E-02	12597.37
6	33		3.77E-02	12597.32
23	33		4.16E-02	12597.52
5	33		4.20E-02	12597.54
14	33		4.48E-02	12597.84
33	41		4.88E-02	12597.84
**3 SNPs at a time**				
3	26	33	3.09E-04	12586.74
5	26	33	2.78E-03	12591.74
16	30	41	4.07E-03	12592.65
1	3	33	4.23E-03	12592.63
3	27	33	6.53E-03	12593.79
4	15	33	7.42E-03	12594.10
9	16	33	7.59E-03	12594.15
1	8	39	7.88E-03	12594.52
2	15	33	8.48E-03	12594.23
2	16	33	9.07E-03	12594.38
16	30	40	9.30E-03	12594.64
16	33	41	9.40E-03	12594.67
9	14	33	9.62E-03	12594.73
7	15	33	9.84E-03	12594.78
2	17	33	9.86E-03	12594.58
