# Supplementary material for: Evaluation of 41 Candidate Gene Variants for Obesity in the EPIC-Potsdam Cohort by Multi-Locus Stepwise Regression
Source: PLoS One. 2013 Jul 12;8(7):e68941. doi: 10.1371/journal.pone.0068941 (PMC3709896; doi:10.1371/journal.pone.0068941)
Supplement: Table S9 — All other associations of the MSR-selected SNP combinations with obesity-related traits (BMI, waist circumference and waist-circumference adjusted for BMI) in 2,122 European middle-aged men and women (random population sample). (PDF) [file pone.0068941.s011.pdf]

Table S9: All other associations of the MSR-selected SNP combinations with obesity-related traits (BMI, waist circumference and waist-circumference adjusted for BMI) in 2,122 European middle-aged men and women (random population sample).

Outcome global p-value (F test) (permutation p-value)[a]	SNPs [b]	allele combination [c]	Frequency	Beta (SE) [d]	p-value
**BMI (kg/m2)**	7-13-15-18-29-31	112111	0.088	-0.38 (0.28)	1.77E-01
p= 7.53E-06 (0.515)		111211	0.097	-0.08 (0.27)	7.78E-01
		111111	0.126	0.65 (0.23)	3.99E-03
		112211	0.100	0.83 (0.27)	1.87E-03
**BMI (kg/m2)**	4-19-26-36-37-41	112111	0.064	-1.58 (0.33)	2.03E-06
p= 7.53E-06 (0.515)		121111	0.060	-0.60 (0.34)	8.00E-02
		111111	0.161	-0.18 (0.21)	3.95E-01
		111121	0.128	0.12 (0.23)	5.86E-01
**BMI (kg/m2)**	23-26-33-36-37-40	121111	0.072	-1.52 (0.30)	5.76E-07
p= 8.18E-06 (0.545)		111111	0.213	-0.26 (0.17)	1.41E-01
		111121	0.157	0.04 (0.20)	8.35E-01
		111112	0.058	0.49 (0.33)	1.46E-01
**BMI (kg/m2)**	1-7-13-15-18-29	111211	0.089	-0.39 (0.27)	1.54E-01
p= 8.21E-06 (0.545)		111121	0.100	-0.10 (0.26)	6.98E-01
		111221	0.105	0.71 (0.26)	6.09E-03
		111111	0.122	0.74 (0.23)	1.39E-03
**BMI (kg/m2)**	11-23-26-33-37-40	112111	0.074	-1.35 (0.30)	5.67E-06
p= 8.50E-06 (0.545)		111111	0.226	-0.42 (0.17)	1.32E-02
		111121	0.159	0.07 (0.20)	7.43E-01
		111112	0.064	0.53 (0.31)	9.12E-02
**BMI (kg/m2)**	4-17-26-36-37-40	112111	0.058	-1.77 (0.36)	8.18E-07
p= 9.47E-06 (0.570)		121111	0.070	-0.48 (0.31)	1.24E-01
		111111	0.162	-0.21 (0.20)	3.06E-01
		121121	0.054	-0.03 (0.37)	9.38E-01
.		111121	0.125	0.12 (0.23)	6.13E-01
**BMI (kg/m2)**	1-13-15-18-29-38	112111	0.069	-0.43 (0.31)	1.67E-01
p= 9.70E-06 (0.580)		111211	0.078	-0.42 (0.30)	1.61E-01
		111111	0.098	0.35 (0.27)	1.93E-01
		112212	0.052	0.59 (0.38)	1.19E-01
		112211	0.078	0.62 (0.31)	4.55E-02
		111112	0.056	1.54 (0.38)	4.42E-05
**WC adj. BMI (cm)**	16-30-41	221	0.057	-0.43 (0.37)	2.49E-01
p= 7.31E-03 (0.990)		211	0.329	-0.30 (0.16)	6.53E-02
		111	0.321	0.03 (0.16)	8.64E-01
		112	0.102	0.05 (0.27)	8.64E-01
		212	0.097	0.39 (0.28)	1.64E-01
		121	0.060	1.12 (0.36)	2.20E-03
**WC adj. BMI (cm)**	1-3-33	112	0.085	-0.86 (0.29)	3.00E-03
p= 4.23E-03 (0.990)		211	0.227	-0.24 (0.18)	1.78E-01
		121	0.142	0.09 (0.21)	6.64E-01
		111	0.488	0.38 (0.15)	1.30E-02
**WC adj. BMI (cm)**	3-27-33	112	0.063	-1.17 (0.34)	6.34E-04
p= 1.02E-02 (0.990)		122	0.058	-0.28 (0.36)	4.42E-01
		121	0.306	-0.07 (0.17)	6.89E-01
		211	0.085	-0.02 (0.29)	9.55E-01
		111	0.409	0.27 (0.15)	8.52E-02
		221	0.058	0.37 (0.38)	3.30E-01
**WC adj. BMI (cm)**	4-15-33	122	0.054	-1.10 (0.38)	3.84E-03
p= 1.08E-02 (0.990)		112	0.064	-0.47 (0.34)	1.71E-01
		211	0.076	-0.18 (0.32)	5.66E-01
		111	0.400	-0.13 (0.16)	4.04E-01
		121	0.314	0.35 (0.17)	3.86E-02
		221	0.068	0.72 (0.35)	3.84E-02
**WC adj. BMI (cm)**	9-16-33	121	0.292	-0.26 (0.17)	1.27E-01
p= 7.59E-03 (0.990)		122	0.052	-0.15 (0.39)	7.10E-01
		221	0.135	0.20 (0.24)	4.14E-01

Outcome<br>global p-value (F test)<br>(permutation p-value)[a]	SNPs [b]	allele combination [c]	Frequency	Beta (SE) [d]	p-value
		111	0.294	0.24 (0.17)	1.63E-01
		211	0.137	0.51 (0.24)	3.80E-02
**WC adj. BMI (cm)**	1-8-39	212	0.068	-1.26 (0.36)	5.88E-04
p= 1.26E-02 (0.990)		121	0.162	-0.22 (0.23)	3.25E-01
		221	0.054	-0.13 (0.42)	7.62E-01
		122	0.114	0.04 (0.27)	8.79E-01
		211	0.099	0.12 (0.29)	6.80E-01
		112	0.196	0.21 (0.21)	3.08E-01
		111	0.263	0.27 (0.18)	1.40E-01
**WC adj. BMI (cm)**	2-15-33	211	0.221	-0.33 (0.19)	8.36E-02
p= 8.48E-03 (0.990)		111	0.255	0.07 (0.18)	7.16E-01
		221	0.169	0.09 (0.22)	6.70E-01
		121	0.214	0.65 (0.20)	1.22E-03
**WC adj. BMI (cm)**	2-16-33	221	0.184	-0.27 (0.21)	2.06E-01
p= 9.07E-03 (0.990)		211	0.206	-0.04 (0.20)	8.55E-01
		121	0.244	0.02 (0.19)	8.94E-01
		111	0.225	0.64 (0.19)	8.41E-04
**WC adj. BMI (cm)**	16-30-40	221	0.057	-0.39 (0.37)	2.85E-01
p= 1.59E-02 (0.990)		211	0.338	-0.29 (0.16)	7.21E-02
		111	0.332	0.01 (0.16)	9.74E-01
		112	0.091	0.12 (0.28)	6.66E-01
		212	0.087	0.42 (0.30)	1.62E-01
		121	0.063	1.02 (0.35)	4.08E-03
**WC adj. BMI (cm)**	16-33-41	121	0.052	-0.81 (0.38)	3.53E-02
p= 1.79E-02 (0.990)		221	0.057	-0.57 (0.36)	1.16E-01
		211	0.329	-0.27 (0.16)	9.68E-02
		112	0.101	0.11 (0.28)	6.93E-01
		212	0.098	0.38 (0.28)	1.71E-01
		111	0.329	0.40 (0.16)	1.42E-02
**WC adj. BMI (cm)**	9-14-33	112	0.066	-0.10 (0.33)	7.60E-01
p= 9.62E-03 (0.990)		121	0.218	-0.09 (0.19)	6.40E-01
		111	0.369	0.04 (0.16)	8.00E-01
		221	0.095	0.08 (0.29)	7.86E-01
		211	0.176	0.50 (0.21)	1.84E-02
**WC adj. BMI (cm)**	7-15-33	122	0.057	-0.82 (0.37)	2.76E-02
p= 1.87E-02 (0.995)		211	0.087	-0.56 (0.30)	6.47E-02
		112	0.061	-0.44 (0.35)	2.12E-01
		111	0.388	-0.02 (0.16)	8.87E-01
		121	0.321	0.40 (0.17)	1.65E-02
		221	0.061	0.53 (0.38)	1.60E-01
**WC adj. BMI (cm)**	2-17-33	221	0.116	-0.21 (0.27)	4.40E-01
p= 9.86E-03 (0.995)		121	0.134	-0.15 (0.24)	5.48E-01
		211	0.274	-0.12 (0.18)	4.88E-01
		111	0.334	0.55 (0.16)	7.71E-04

BMI=Body-mass index, WC=waist circumference, SE=Standard error

[a] Permutation test based on 200 permutations.

[b] SNP names and characteristics could be found in Table 2.

[c] The major allele was coded as 1 and the minor one as 2.

[d] Association results are based on models adjusted for sex, age, education, occupational activity, sports activity, smoking, and alcohol intake, energy intake, fat intake, and fruit and vegetable intake.
