# Supplementary material for: Evaluation of 41 Candidate Gene Variants for Obesity in the EPIC-Potsdam Cohort by Multi-Locus Stepwise Regression
Source: PLoS One. 2013 Jul 12;8(7):e68941. doi: 10.1371/journal.pone.0068941 (PMC3709896; doi:10.1371/journal.pone.0068941)

**Figure S2: LD plot for TBC1D1 gene of the EPIC Potsdam subsample (N=2,122).** Disequilibrium coefficient r² values were generated using Haploview version 4.2 (Barrett JC et al. Bioinformatics 2005;21(2):263-5). Standard Color Scheme for the LD plots for r² were used (for more information see Haploview documentation).

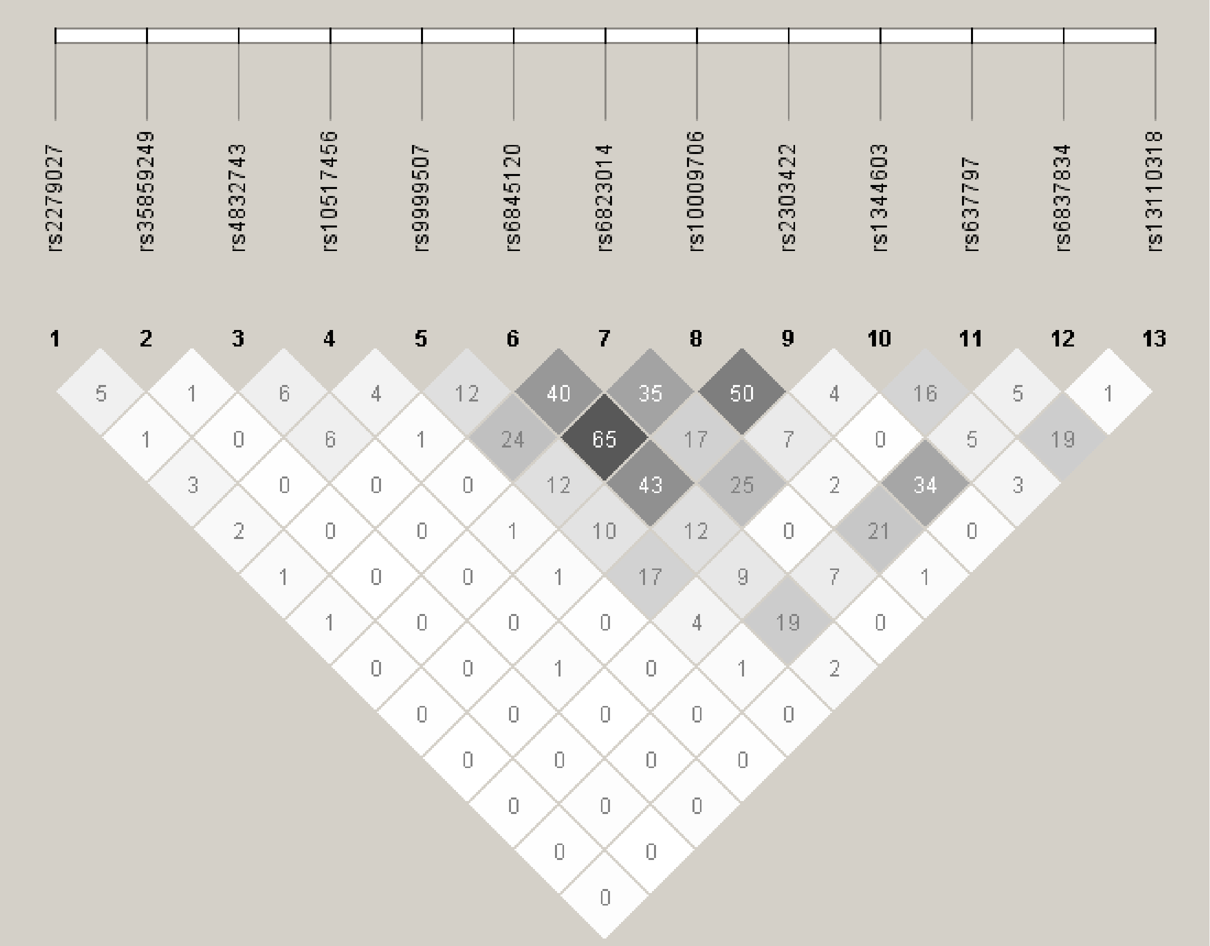

Supplement: Figure S2 — LD plot for TBC1D1 gene of the EPIC Potsdam subsample (N = 2,122). Disequilibrium coefficient r2 values were generated using Haploview version 4.2 (Barrett JC et al. Bioinformatics 2005;21(2):263-5). Standard Color Scheme for the LD plots for r2 were used (for more information see Haploview documentation). (PDF) [file pone.0068941.s002.pdf]
