# Supplementary material for: Evaluation of 41 Candidate Gene Variants for Obesity in the EPIC-Potsdam Cohort by Multi-Locus Stepwise Regression
Source: PLoS One. 2013 Jul 12;8(7):e68941. doi: 10.1371/journal.pone.0068941 (PMC3709896; doi:10.1371/journal.pone.0068941)
Supplement: Text S1 — Physiological mechanisms of selected genes in our study. (PDF) [file pone.0068941.s012.pdf]

Text S1. Physiological mechanisms of selected genes in our study

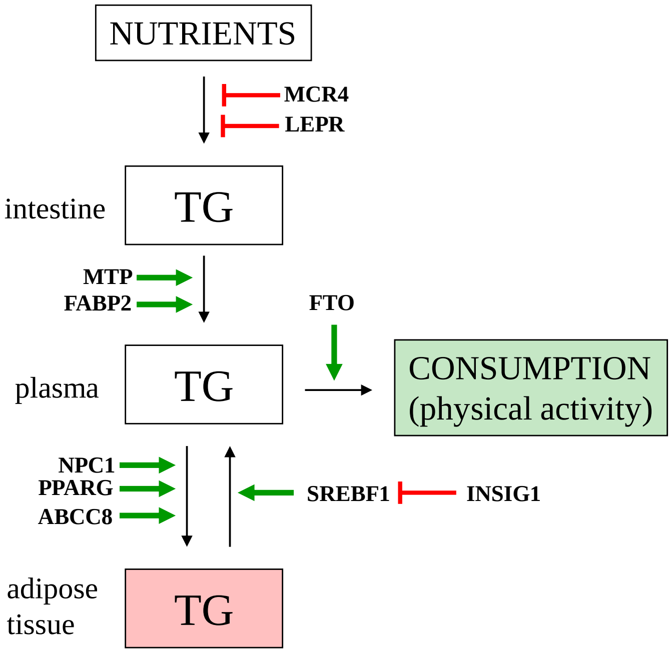

Figure 3: Physiological mechanisms

Description of the physiological effects of the selected genes on obesity

INSIG2 blocks lipid synthesis by preventing the translocation of the transcription factors to the nucleus and transcription of target genes declines. The triacylglycerol reducing effect of fibrates and thiazolidinedionesis partially accounted for by inhibition of SREBP-1 activation via up-regulation of Insig [1]

FTO (fat mass and obesity-associated protein) is also known as alpha-ketoglutarate-dependent dioxygenase. It is speculated that a yet unidentified transcript of the common FTO gene promotes lipolysis in adipocytes or, alternatively, exerts an anti-lipogenic effect indirectly through mechanisms such as appetite/satiety, endocrine, automatic nervous system, or exercise tolerance [2]

MC4R is a G-protein coupled receptor. Constitutive endocytosis of this receptor is essential to maintain receptor responsiveness to α-Melanocyte-stimulating Hormone (α-MSH). Deficiency of this receptor impairs hypothalamic secretion of hormones signalling satiety [3]

NPC1 (Niemann-Pick C1-like protein 1) regulates the intracellular transport of endocytosed lipids ingested by endocytosis. It promotes the storage of triglycerides in adipocytes [4]

MTP encodes the large subunit of the heterodimeric microsomal triglyceride transfer protein, a central regulator of microsomal lipoprotein assembly. It thus favours the release of lipids from the intestine to the circulation [5]

SREBF1 encodes the transcription factor SREB-1a/c regulates being central hubs in the cellular lipid metabolism. Overexpression of SREB-1a in mouse adiopcytes results in hypertrophy [6]

LEPR – leptin receptor binds the hormone leptin which is secreted by adipose cells and in the brain inhibits the liberation of the orexigenic peptides like peptide Y. Deficiency of the receptor results in a preponderance of orexigenic signals [7]

PPAR-gamma is a pro-lipogenic transcription factor which is essential for the proliferation of adipose tissue. A deficiency of this transcription factor is associated with a reduced size of adipose tissue [8]

FABP1/2 denotes cellular fatty acid binding proteins. In intestinal cells they transport free fatty acids to the re-esterifying enzymes and thus contribute to an efficient transfer of lipids from the intestinal lumen to the blood [9]

ABCC8 This ATP-binding cassette transporter sub-family C member 8 regulates insulin secretion in ß cells [10]. Insulin promotes uptake of free fatty acids into adipocytes and their esterification to TG.

1. Konig, B., Koch, A., Spielmann, J., Hilgenfeld, C., Hirche, F., Stangl, G. I. & Eder, K. (2009) Activation of PPARalpha and PPARgamma reduces triacylglycerol synthesis in rat hepatoma cells by reduction of nuclear SREBP-1, European journal of pharmacology. 605, 23-30.
2. Wahlen, K., Sjolin, E. & Hoffstedt, J. (2008) The common rs9939609 gene variant of the fat mass- and obesity-associated gene FTO is related to fat cell lipolysis, Journal of lipid research. 49, 607-11.
3. Branson, R., Potoczna, N., Kral, J. G., Lentes, K. U., Hoehe, M. R. & Horber, F. F. (2003) Binge eating as a major phenotype of melanocortin 4 receptor gene mutations, The New England journal of medicine. 348, 1096-103.
4. Bambace, C., Dahlman, I., Arner, P. & Kulyte, A. NPC1 in human white adipose tissue and obesity, BMC endocrine disorders. 13, 5.
5. Li, J., Bertinato, P., Cheng, H., Cole, B. M., Bronk, B. S., Jaynes, B. H., Hickman, A., Haven, M. L., Kolosko, N. L., Barry, C. J. & Manion, T. B. (2006) Discovery of potent and orally active MTP inhibitors as potential anti-obesity agents, Bioorganic & medicinal chemistry letters. 16, 3039-42.
6. Horton, J. D., Shimomura, I., Ikemoto, S., Bashmakov, Y. & Hammer, R. E. (2003) Overexpression of sterol regulatory element-binding protein-1a in mouse adipose tissue produces adipocyte hypertrophy, increased fatty acid secretion, and fatty liver, The Journal of biological chemistry. 278, 36652-60.
7. Farooqi, I. S. & O'Rahilly, S. (2009) Leptin: a pivotal regulator of human energy homeostasis, The American journal of clinical nutrition. 89, 980S-984S.
8. Altshuler, D., Hirschhorn, J. N., Klannemark, M., Lindgren, C. M., Vohl, M. C., Nemesh, J., Lane, C. R., Schaffner, S. F., Bolk, S., Brewer, C., Tuomi, T., Gaudet, D., Hudson, T. J., Daly, M., Groop, L. & Lander, E. S. (2000) The common PPARgamma Pro12Ala polymorphism is associated with decreased risk of type 2 diabetes, Nature genetics. 26, 76-80.
9. Levy, E., Menard, D., Delvin, E., Stan, S., Mitchell, G., Lambert, M., Ziv, E., Feoli-Fonseca, J. C. & Seidman, E. (2001) The polymorphism at codon 54 of the FABP2 gene increases fat absorption in human intestinal explants, The Journal of biological chemistry. 276, 39679-84.
10. Campbell, J. D., Sansom, M. S. & Ashcroft, F. M. (2003) Potassium channel regulation, EMBO reports. 4, 1038-42.
